# Identifying organ dysfunction trajectory-based subphenotypes in critically ill patients with COVID-19

**DOI:** 10.1038/s41598-021-95431-7

**Published:** 2021-08-05

**Authors:** Chang Su, Zhenxing Xu, Katherine Hoffman, Parag Goyal, Monika M. Safford, Jerry Lee, Sergio Alvarez-Mulett, Luis Gomez-Escobar, David R. Price, John S. Harrington, Lisa K. Torres, Fernando J. Martinez, Thomas R. Campion, Fei Wang, Edward J. Schenck

**Affiliations:** 1 Department of Population Health Sciences, Weill Cornell Medicine, 425 E 61 St., New York, NY 10065 USA; 2 Division of General Internal Medicine, Joan and Sanford I. Weill Department of Medicine, Weill Cornell Medicine, New York, NY USA; 3 New York-Presbyterian Hospital, Weill Cornell Medicine, 1300 York Ave., Box 96, New York, NY 10065 USA; 4 Weill Cornell Medical College, Weill Cornell Medicine, New York, NY USA; 5 Division of Pulmonary and Critical Care Medicine, Joan and Sanford I. Weill Department of Medicine, Weill Cornell Medicine, New York, NY USA

**Keywords:** Respiration, Viral infection, Prognosis

## Abstract

COVID-19-associated respiratory failure offers the unprecedented opportunity to evaluate the differential host response to a uniform pathogenic insult. Understanding whether there are distinct subphenotypes of severe COVID-19 may offer insight into its pathophysiology. Sequential Organ Failure Assessment (SOFA) score is an objective and comprehensive measurement that measures dysfunction severity of six organ systems, i.e., cardiovascular, central nervous system, coagulation, liver, renal, and respiration. Our aim was to identify and characterize distinct subphenotypes of COVID-19 critical illness defined by the post-intubation trajectory of SOFA score. Intubated COVID-19 patients at two hospitals in New York city were leveraged as development and validation cohorts. Patients were grouped into mild, intermediate, and severe strata by their baseline post-intubation SOFA. Hierarchical agglomerative clustering was performed within each stratum to detect subphenotypes based on similarities amongst SOFA score trajectories evaluated by Dynamic Time Warping. Distinct worsening and recovering subphenotypes were identified within each stratum, which had distinct 7-day post-intubation SOFA progression trends. Patients in the worsening suphenotypes had a higher mortality than those in the recovering subphenotypes within each stratum (mild stratum, 29.7% vs. 10.3%, p = 0.033; intermediate stratum, 29.3% vs. 8.0%, p = 0.002; severe stratum, 53.7% vs. 22.2%, p < 0.001). Pathophysiologic biomarkers associated with progression were distinct at each stratum, including findings suggestive of inflammation in low baseline severity of illness versus hemophagocytic lymphohistiocytosis in higher baseline severity of illness. The findings suggest that there are clear worsening and recovering subphenotypes of COVID-19 respiratory failure after intubation, which are more predictive of outcomes than baseline severity of illness. Distinct progression biomarkers at differential baseline severity of illness suggests a heterogeneous pathobiology in the progression of COVID-19 respiratory failure.

## Introduction

The COVID-19 pandemic has created an unprecedented opportunity to explore a large cohort of patients infected with a single pathogen thus providing a window to examine patient variability in response to a uniform insult. Indeed, a number of immunologic studies have sought to understand the disease in terms of clustered phenotypic immune responses^1–6^. SARS-CoV-2 infection often leads to hypoxemic respiratory failure requiring treatment with mechanical ventilation which meets clinical and pathologic criteria for Acute Respiratory Distress Syndrome (ARDS)^7–9^. In COVID-19 respiratory failure, like other forms of ARDS, there is significant risk of morbidity and mortality. However, there is clear heterogeneity in outcomes, even in those treated with mechanical ventilation^7,8,10–12^. The baseline clinical characteristics and predictors of mortality of those requiring mechanical ventilation have been described^7,10,11,13^. Other studies also explored the phenotypes of COVID-19 induced ARDS^4,5^. These studies offer some insight into a differential host response but are limited to characterizing patients at baseline.

In prior studies of ARDS^14,15^, unique subphenotypes have been described, which identify hyperinflammatory and hypoinflammatory populations with differential demographics, clinical characteristics, inflammatory markers and outcomes. These subphenotypes are primarily characterized by host response inflammatory markers and patterns of organ injury, but are agnostic of the type of insult or infection. In COVID-19, baseline risk stratification may be insufficient to characterize subphenotypes that accurately reflect the complexity of the disease arc^16^. Sequential Organ Failure Assessment (SOFA) is a scoring system of tracking patient’s organ dysfunction severity during the stay in intensive care unit (ICU)^17–20^. The SOFA scoring system comprehensively evaluates organ failure from six organ systems, including cardiovascular, central nervous system, coagulation, liver, renal, and respiration. Previous studies have demonstrated that SOFA is a good indicator of outcome (e.g., mortality) of critically ill patients in ICU^20,21^. Serial, temporally ordered, SOFA and comprehensive Electronic Health Records (EHR) data are well suited to develop data-driven subphenotypes^22,23^, where the goal is to identify coherent patient groups with similar clinical courses. Dynamic time warping (DTW)^24^ is a well-established machine learning algorithm for evaluating the similarities among temporal sequences^25,26^. DTW is particularly well suited to evaluate longitudinal changes in organ dysfunction in COVID-19. Characterizing a more complete representation of the disease course in COVID-19 may offer insight into its pathophysiology.

We conducted a two staged post-intubation trajectory analysis of SOFA-based organ dysfunction in patients with COVID-19 to identify unique subphenotypes: Patients were first grouped into mild, intermediate, and severe strata by their baseline SOFA scores; then hierarchical agglomerative clustering was performed within each stratum to detect subphenotypes based on similarities amongst SOFA score trajectories evaluated by DTW. In order to understand the differential disease course, we then explored clinical and biologic features including demographics, comorbidities, clinical characteristics, inflammatory markers, and treatments predictive of these trajectories.

## Methods

### Study design and cohort description

We used individual patient data from two New York Presbyterian (NYP) system hospitals located in New York city: the New York Presbyterian Hospital-Weill Cornell Medical Center (NYP-WCMC), an 862-bed quaternary care hospital, and the New York Presbyterian-Lower Manhattan Hospital (NYP-LMH), a 180-bed non-teaching academic affiliated hospital. Patients were admitted from Mar 3, 2020 to May 12, 2020. SARS-CoV2 diagnosis was made through reverse-transcriptase–PCR assays performed on nasopharyngeal swabs. The critical care response to the pandemic has been previously described^27^. The NYP-WCMC cohort was used as the development cohort to derive subphenotypes, and the NYP-LMH cohort was used for validation. The focus of this study was critically ill patients with COVID-19 who were treated with intubation (Supplementary Appendix 1).

### Data collection

We collected all data from either the Weill Cornell-Critical carE Database for Advanced Research (WC-CEDAR), Weill Cornell Medicine COVID Institutional Data Repository (COVID-IDR), or via manual chart abstraction (REDCap). WC-CEDAR aggregates and transforms data from institutional electronic health records for all patients treated in ICUs in NYP-WCMC and NYP-LMH^28,29^. The COVID-IDR contains additional aggregate EHR data on all patients who were tested for SARS-CoV-2 at NYP-WCMC or NYP-LMH. The REDCap database contains high-quality manually abstracted data on all patients who tested positive for COVID-19 at NYP-WCMC or NYP-LMH^30^. In our analysis, the patient information incorporated included demographics, laboratory tests, vital signs, and respiratory variables obtained from WC-CEDAR, comorbidity information obtained from the REDCap database, and medication data obtained from the COVID-IDR. Data analyzed included demographics, comorbidities, prescribed medications, laboratory test values, vital signs, and respiratory variables. Laboratory test values (e.g., albumin level), vital signs (e.g., temperature), respiratory variables (e.g., PaO_2_/FiO_2_ ratio) were collected daily, and the average value was taken if more than one result was recorded on a given day. All patient characteristics and clinical variables analyzed were detailed in Supplementary Appendix 2 and Supplementary Table S1.

### SOFA calculation

The SOFA score is the sum of six organ dysfunction subscores, including cardiovascular, central nervous system (CNS), coagulation, liver, renal, and respiration^17,20^. In this study, the CNS, coagulation, liver, and renal subscores were derived according to the standard SOFA scoring system^17^. The respiration subscore was calculated using a combination of the traditional and modified scoring method^31^. The cardiovascular SOFA subscore was calculated with additional vasopressors according to a norepinephrine equivalency table, where phenylephrine and vasopressin were converted to a norepinephrine equivalency^32^. SOFA scores were derived every 24 h from the time of intubation, and the worst score within that 24-h data period was selected for each patient^17^.

### Inclusion exclusion criteria

We included patients with positive results on viral RNA detection by real-time reverse transcriptase polymerase chain reaction (RT-PCR) test from nasopharyngeal swabs specimens and treated with mechanical ventilation at the ICU in NYP-WCM and NYP-LMH. We excluded patients who were less than 18 years old. Since our aim was to identify clinically meaningful organ dysfunction progression patterns of intubated patients, trajectories with low quality [20 (5.7%) patients missing over 50% SOFA records] and outlier trajectories [10 (2.9%) patients with unchanged or heavily fluctuated SOFA trajectories within the 7-day window after intubation] were excluded from the analysis (Supplementary Appendix 3 and Supplementary Fig. S1).

### Subphenotype identification

SOFA scores were derived every 24 h and post intubation 7-day SOFA trajectories were constructed for analysis. Missing values within a trajectory were imputed based on the last observation carried forward (LOCF) strategy.

**Figure 1:**
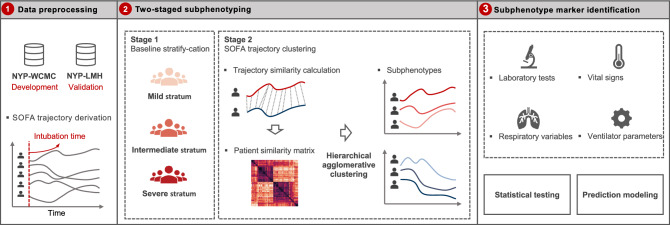
A schematic of the analysis plan. Intubated patients of two cohorts, New NYP-WCMC and NYP-LMH cohorts were analyzed, as development and validation cohorts, respectively. 7-day post-intubation SOFA trajectories were constructed. A two-stage subphenotyping model was then performed on the top of the SOFA trajectories. Statistical testing and prediction modeling were finally performed to identify markers at early stage after intubation for separating the identified trajectory subphenotypes. *NYP-WCMC* New York Presbyterian Hospital-Weill Cornell Medical Center, *NYP-LMH* New York Presbyterian-Lower Manhattan Hospital, *SOFA* Sequential Organ Failure Assessment.

A two-staged subphenotyping method was performed to derive SOFA trajectory subphenotypes (Fig. 1). In the first stage, we used baseline SOFA to group patients with a similar upfront risk of death^20^, as additive organ dysfunction has previously been identified to be associated with poor outcomes in COVID19^11^. We partitioned the patients into three baseline severity strata (mild, intermediate, and severe) according to their SOFA scores within the first 24 h after intubation. The SOFA score cut-offs were set to 0–10, 11–12, and 13–24 in order to: (1) achieve clinically and biologically meaningful strata that have distinct organ dysfunction patterns at baseline (the time of intubation); and (2) obtain a balanced distribution of patients across the three strata. In the second stage, we identified the subphenotypes with similar 7-day SOFA progression patterns. Dynamic Time Warping (DTW)^24^ was adopted to evaluate the similarities between pairwise patient SOFA trajectories within each baseline stratum and then hierarchical agglomerative clustering (HAC)^33^ was performed on these similarities to derive the similar patient clusters as trajectory subphenotypes. DTW can account for the differences among the evolution heterogeneity among the temporal curves and is thus able to evaluate their similarity more robustly^24^. The optimal numbers of subphenotypes were determined by clear separation illustrated by clustergram according to the McClain index^34^.

To validate these findings, we replicated these subphenotypes from the NYP-LMH cohort.

### Clinical outcomes

We analyzed 30-day all-cause mortality as the primary outcome for patients within each phenotype. Successful extubation or need for tracheostomy within 30 days after intubation were secondary outcomes.

### Statistical methods

We characterized the identified subphenotypes by demographics, comorbidities, medications and blood types. We also assessed the 7-day post-intubation trajectories in terms of each clinical variable (including laboratory test values, vital signs, and respiratory variables) among the subphenotypes.

Univariate statistical tests were performed in those association analyses. Specifically, one-way analysis of variance (ANOVA, with Tukey HSD post hoc test), Kruskal–Wallis test (with Dunn post hoc test), student’s t-test, Mann–Whitney test, Chi-square test, and Fisher’s exact test have been used whenever appropriate. The p-values were then corrected for multiple testing using false discovery rate (FDR) estimation. Analysis of covariance (ANCOVA) for the between-strata/subphenotypes comparisons was also applied based on the generalized linear model (GLM) with adjustment on age at baseline.

### Subphenotype prediction modeling

We trained a random forest model with the trajectory subphenotypes as targets and the patient clinical characteristics at specific time points after intubation as input predictors to define if these trajectory subphenotypes can be predicted early. Candidate predictors included demographics, comorbidities, medications prescribed around the intubation event, SOFA subscores, laboratory tests, vital signs, and respiratory variables as described above. The models were calibrated under fivefold cross validation strategy. Prediction performances were measured by area under the receiver operating characteristics (AUC-ROC) and the area under the precision recall curve (AUC-PR). The importance of predictors was visualized as a heatmap to demonstrate their contributions on subphenotype prediction.

### Statement

The IRB of Weill Cornell Medicine approved this study (protocol number 20-04021909) and issued a waiver of informed consent since all examinations were part of standard patient care. We confirm that all research was performed in accordance with relevant regulations. All methods performed in this analysis were in accordance with the declaration of Helsinki and all relevant guidelines and regulations.

## Results

### Patients and baseline severity strata

Inclusion and exclusion criteria are described in Supplementary Fig. S1. A total of 318 mechanically ventilated COVID-19 patients from the New York Presbyterian Hospital-Weill Cornell Medical Center (NYP-WCMC) cohort were included for analysis, consisting of 100 females (31.45%) and an average age of 62.78 $\pm$ 14.34. One day post-intubation the mean SOFA score for this cohort is 11.89 $\pm$ 2.56. A total of 84 mechanically ventilated COVID-19 patients from the New York Presbyterian-Lower Manhattan Hospital (NYP-LMH) were included as a validation cohort, consisting of 33 (39.29%) females and an average age of 66.06 $\pm$ 13.06. One day post-intubation the mean SOFA score is 12.51 $\pm$ 2.25. The clinical characteristics of both cohorts are summarized in Table 1.

**Table 1:** Clinical characteristics of the studied cohorts.

Variable	NYP-WCMC cohort				NYP-LMH validation cohort			
	All	Mild stratum	Intermediate stratum	Severe stratum	All	Mild stratum	Intermediate stratum	Severe stratum
# of patients (%)	318	76 (23.90)	116 (36.48)	126 (39.62)	84	10 (11.90)	35 (41.67)	39 (46.43)
**Demographics**								
Age, mean (SD)	62.78 (14.34)	61.47 (16.51)	60.53 (14.14)	65.64 (12.52)	66.06 (13.06)	61.00 (17.10)	61.63 (11.46)	71.33 (11.07)
Sex female, n (%)	100 (31.45%)	23 (30.26%)	38 (32.76%)	39 (30.95%)	33 (39.29%)	4 (40.00%)	19 (54.29%)	10 (25.64%)
Caucasian, n (%)	91 (28.62%)	20 (26.32%)	39 (33.62%)	32 (25.40%)	7 (8.33%)	0 (0.00%)	4 (11.43%)	3 (7.69%)
African American, n (%)	27 (8.49%)	3 (3.95%)	5 (4.31%)	19 (15.08%)	7 (8.33%)	0 (0.00%)	0 (0.00%)	7 (17.95%)
Asian/Pacific Islander, n (%)	33 (10.38%)	11 (14.47%)	9 (7.76%)	13 (10.32%)	32 (38.10%)	5 (50.00%)	12 (34.29%)	15 (38.46%)
Multi-racial, n (%)	86 (27.04%)	21 (27.63%)	34 (29.31%)	31 (24.60%)	10 (11.90%)	2 (20.00%)	5 (14.29%)	3 (7.69%)
BMI, mean (SD)	29.53 (8.40)	29.23 (9.06)	30.75 (9.17)	28.59 (7.01)	28.70 (7.70)	26.67 (3.94)	30.03 (9.94)	28.03 (5.61)
**Comorbidities**								
Coronary artery disease, n (%)	49 (15.41%)	7 (9.21%)	17 (14.66%)	25 (19.84%)	11 (13.10%)	1 (10.00%)	1 (2.86%)	9 (23.08%)
Cerebrovascular accident (stroke), n (%)	20 (6.29%)	3 (3.95%)	7 (6.03%)	10 (7.94%)	4 (4.76%)	0 (0.00%)	0 (0.00%)	4 (10.26%)
Heart failure, n (%)	21 (6.60%)	3 (3.95%)	9 (7.76%)	9 (7.14%)	3 (3.57%)	0 (0.00%)	1 (2.86%)	2 (5.13%)
Hypertension, n (%)	167 (52.52%)	35 (46.05%)	57 (49.14%)	75 (59.52%)	50 (59.52%)	5 (50.00%)	17 (48.57%)	28 (71.79%)
Diabetes mellitus, n (%)	94 (29.56%)	17 (22.37%)	30 (25.86%)	47 (37.30%)	35 (41.67%)	4 (40.00%)	12 (34.29%)	19 (48.72%)
Pulmonary disease, n (%)	63 (19.81%)	15 (19.74%)	22 (18.97%)	26 (20.63%)	15 (17.86%)	2 (20.00%)	4 (11.43%)	9 (23.08%)
Renal disease, n (%)	26 (8.18%)	5 (6.58%)	5 (4.31%)	16 (12.70%)	7 (8.33%)	0 (0.00%)	2 (5.71%)	5 (12.82%)
Cirrhosis, n (%)	5 (1.57%)	3 (3.95%)	0 (0.00%)	2 (1.59%)	1 (1.19%)	0 (0.00%)	0 (0.00%)	1 (2.56%)
Hepatitis, n (%)	4 (1.26%)	1 (1.32%)	0 (0.00%)	3 (2.38%)	2 (2.38%)	0 (0.00%)	1 (2.86%)	1 (2.56%)
HIV, n (%)	4 (1.26%)	1 (1.32%)	2 (1.72%)	1 (0.79%)	1 (1.19%)	0 (0.00%)	1 (2.86%)	0 (0.00%)
Active cancer, n (%)	21 (6.60%)	3 (3.95%)	2 (1.72%)	16 (12.70%)	2 (2.38%)	0 (0.00%)	0 (0.00%)	2 (5.13%)
Transplant, n (%)	14 (4.40%)	5 (6.58%)	3 (2.59%)	6 (4.76%)	1 (1.19%)	0 (0.00%)	0 (0.00%)	1 (2.56%)
Inflammatory bowel disease, n (%)	7 (2.20%)	2 (2.63%)	2 (1.72%)	3 (2.38%)	0 (0.00%)	0 (0.00%)	0 (0.00%)	0 (0.00%)
Rheumatologic disease, n (%)	15 (4.72%)	4 (5.26%)	3 (2.59%)	8 (6.35%)	3 (3.57%)	0 (0.00%)	2 (5.71%)	1 (2.56%)
Other immunosuppressed state, n (%)	12 (3.77%)	4 (5.26%)	1 (0.86%)	7 (5.56%)	0 (0.00%)	0 (0.00%)	0 (0.00%)	0 (0.00%)
**Baseline SOFA scores**								
Cardiovascular, mean (SD)	3.02 (1.35)	1.32 (1.34)	3.41 (0.88)	3.69 (0.70)	3.45 (1.03)	1.40 (1.02)	3.57 (0.80)	3.87 (0.40)
Central nervous system, mean (SD)	3.72 (0.68)	3.34 (1.13)	3.72 (0.47)	3.94 (0.24)	3.39 (0.74)	2.60 (1.36)	3.37 (0.48)	3.62 (0.54)
Coagulation, mean (SD)	0.15 (0.47)	0.12 (0.40)	0.04 (0.20)	0.28 (0.64)	0.13 (0.40)	0.00 (0.00)	0.11 (0.40)	0.18 (0.45)
Liver, mean (SD)	0.24 (0.56)	0.20 (0.46)	0.14 (0.43)	0.37 (0.67)	0.20 (0.48)	0.10 (0.30)	0.14 (0.42)	0.28 (0.55)
Renal, mean (SD)	0.94 (1.32)	0.16 (0.54)	0.35 (0.67)	1.96 (1.44)	1.36 (1.35)	0.50 (0.67)	0.37 (0.64)	2.46 (1.08)
Respiration, mean (SD)	3.81 (0.58)	3.45 (0.89)	3.89 (0.45)	3.97 (0.25)	3.98 (0.22)	4.00 (0.00)	3.94 (0.33)	4.00 (0.00)
SOFA score, mean (SD)	11.89 (2.56)	8.58 (1.84)	11.55 (0.58)	14.20 (1.46)	12.51 (2.25)	8.60 (2.11)	11.51 (0.50)	14.41 (1.08)
**30-Day clinical outcomes**								
Extubation, n (%)	138 (43.40%)	40 (52.63%)	54 (46.55%)	44 (34.92%)	31 (36.90%)	2 (20.00%)	15 (42.86%)	14 (35.90%)
Mortality, n (%)	77 (24.21%)	14 (18.42%)	18 (15.52%)	45 (35.71%)	26 (30.95%)	4 (40.00%)	8 (22.86%)	14 (35.90%)
Tracheostomy, n (%)	41 (12.89%)	10 (13.16%)	18 (15.52%)	13 (10.32%)	6 (7.14%)	0 (0.00%)	3 (8.57%)	3 (7.69%)

*BMI* Body mass index, *HIV* human immunodeficiency virus, *NYP-WCMC* New York Presbyterian Hospital-Weill Cornell Medical Center, *NYP-LMH* New York Presbyterian-Lower Manhattan Hospital, *SD* standard deviation, *SOFA* Sequential Organ Failure Assessment.

For the NYP-WCMC cohort, patients were first partitioned into mild, intermediate, and severe strata based on the SOFA scores within one day after intubation, consisting of 76 (23.29%), 116 (36.48%), and 126 (39.62%) patients, respectively; while for the NYP-LMH validation cohort, the three strata consist of 10 (11.90%), 35 (41.67%), and 39 (46.43%) patients, respectively. As shown in Table 1, the patients in both NYP-WCMC and NYP-LMH cohorts exhibit additive patterns of post intubation baseline organ dysfunction according to the SOFA subscores. Specifically, CNS and respiration dysfunction were present in the mild stratum; the intermediate stratum had additional cardiovascular dysfunction on top of CNS and respiratory dysfunction compared to the mild stratum; and the severe stratum had renal dysfunction in addition to all other organ failure. Liver and coagulation dysfunction were rare in all strata. Patients in the severe stratum were generally older and were more likely to suffer from chronic comorbidities at baseline.

### SOFA trajectory subphenotypes

**Figure 2:**
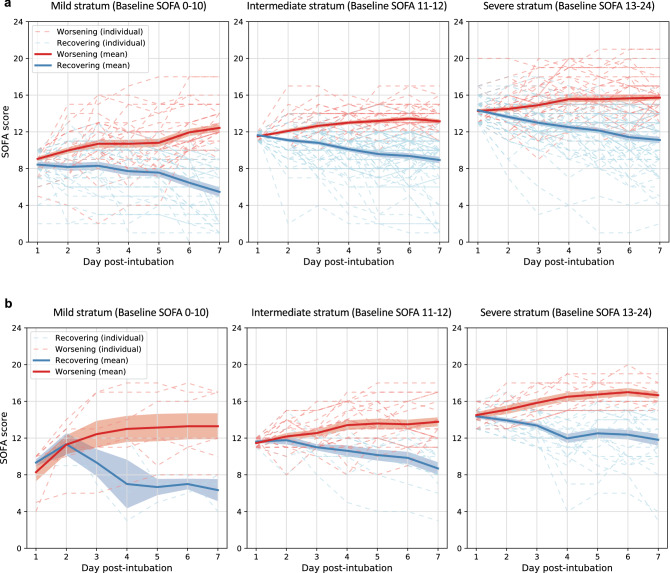
Averaged Sequential Organ Failure Assessment (SOFA) trajectories of the identified subphenotypes. (**a**) SOFA trajectories of subphenotypes derived in NYP-WCMC cohort. (**b**) SOFA trajectories of subphenotypes derived in NYP-LMH validation cohort. Solid curves are mean SOFA trajectories of the subphenotypes, while shadow represents 95% confidence interval. Dashed curves are individual SOFA trajectories of the patients.

The clustergrams built upon the pairwise SOFA trajectory distance matrix derived by DTW are shown in Supplementary Fig. S2. The optimal number of subphenotypes within each stratum as determined by the McClain Index^34^ are shown in Supplementary Table S1, suggesting two being the best choice across all strata in both cohorts. Figure 2 demonstrate the individual averaged SOFA curves for patients in the two subphenotypes across all strata: a worsening subphenotype of which SOFA score increased within the 7-day observation window, and a recovering subphenotype of which SOFA score improved. The clinical characteristics of these subphenotypes were summarized in Table 2. Overall, there was no marked difference in terms of demographics, comorbidity burden, and pattern of organ dysfunction (distribution of SOFA subscores and total score) between the worsening and recovering subphenotypes within each baseline severity stratum at baseline. This suggests that, though the subphenotypes varied in 7-day organ dysfunction progression patterns, they have similar clinical status immediately after intubation. We further investigated medications prescribed within each subphenotype and did not find significant signal as well (Supplementary Table S3). In addition, clinical characteristics and medications of the subphenotypes re-derived in the NYP-LMH validation cohort were summarized in Supplementary Tables S2 and S4.

**Table 2:** Clinical characteristics of the trajectory subphenotypes in NYP-WCMC cohort.

Variable	Mild stratum (SOFA 0–10, n = 76)			Intermediate stratum (SOFA 11–12, n = 116)			Severe stratum (SOFA 13–24, n = 126)		
	Worsening	Recovering	p-value^†^	Worsening	Recovering	p-value^†^	Worsening	Recovering	p-value^†^
Total #	37	39	–	41	75	–	54	72	–
**Demographics**									
Age, mean (SD)	61.08 (14.95)	61.85 (17.86)	0.240	63.80 (13.90)	58.73 (13.95)	0.059	65.72 (11.05)	65.58 (13.52)	0.951
Sex female, n (%)	9 (24.32%)	14 (35.90%)	0.323	13 (31.71%)	25 (33.33%)	1.000	17 (31.48%)	22 (30.56%)	1.000
Caucasian, n (%)	9 (24.32%)	11 (28.21%)	0.927	14 (34.15%)	25 (33.33%)	0.883	16 (29.63%)	16 (22.22%)	0.846
African American, n (%)	1 (2.70%)	2 (5.13%)		2 (4.88%)	3 (4.00%)		8 (14.81%)	11 (15.28%)	
Asian/Pacific Islander, n (%)	5 (13.51%)	6 (15.38%)		3 (7.32%)	6 (8.00%)		4 (7.41%)	9 (12.50%)	
Multi-racial, n (%)	12 (32.43%)	9 (23.08%)		10 (24.39%)	24 (32.00%)		13 (24.07%)	18 (25.00%)	
BMI, mean (SD)	29.42 (10.01)	29.07 (8.21)	0.435	29.99 (7.25)	31.18 (10.09)	0.416	29.79 (7.01)	27.71 (6.89)	0.018
**Comorbidities**									
Coronary artery disease, n (%)	5 (13.51%)	2 (5.13%)	0.248	5 (13.51%)	2 (5.13%)	0.248	11 (20.37%)	14 (19.44%)	0.824
Cerebrovascular accident (stroke), n (%)	0 (0.00%)	3 (7.69%)	0.241	0 (0.00%)	3 (7.69%)	0.241	2 (3.70%)	8 (11.11%)	0.189
Heart failure, n (%)	2 (5.41%)	1 (2.56%)	0.604	2 (5.41%)	1 (2.56%)	0.604	4 (7.41%)	5 (6.94%)	1.000
Hypertension, n (%)	15 (40.54%)	20 (51.28%)	0.479	15 (40.54%)	20 (51.28%)	0.479	35 (64.81%)	40 (55.56%)	0.248
Diabetes mellitus, n (%)	6 (16.22%)	11 (28.21%)	0.275	6 (16.22%)	11 (28.21%)	0.275	24 (44.44%)	23 (31.94%)	0.130
Pulmonary disease, n (%)	7 (18.92%)	8 (20.51%)	1.000	7 (18.92%)	8 (20.51%)	1.000	14 (25.93%)	12 (16.67%)	0.184
Renal disease, n (%)	0 (0.00%)	5 (12.82%)	0.055	0 (0.00%)	5 (12.82%)	0.055	8 (14.81%)	8 (11.11%)	0.589
Cirrhosis, n (%)	1 (2.70%)	2 (5.13%)	1.000	1 (2.70%)	2 (5.13%)	1.000	1 (1.85%)	1 (1.39%)	1.000
Hepatitis, n (%)	0 (0.00%)	1 (2.56%)	1.000	0 (0.00%)	1 (2.56%)	1.000	2 (3.70%)	1 (1.39%)	0.572
HIV, n (%)	0 (0.00%)	1 (2.56%)	1.000	0 (0.00%)	1 (2.56%)	1.000	0 (0.00%)	1 (1.39%)	1.000
Active cancer, n (%)	2 (5.41%)	1 (2.56%)	0.604	2 (5.41%)	1 (2.56%)	0.604	10 (18.52%)	6 (8.33%)	0.102
Transplant, n (%)	1 (2.70%)	4 (10.26%)	0.359	1 (2.70%)	4 (10.26%)	0.359	5 (9.26%)	1 (1.39%)	0.081
Inflammatory bowel disease, n (%)	0 (0.00%)	2 (5.13%)	0.494	0 (0.00%)	2 (5.13%)	0.494	1 (1.85%)	2 (2.78%)	1.000
Rheumatologic disease, n (%)	0 (0.00%)	4 (10.26%)	0.116	0 (0.00%)	4 (10.26%)	0.116	3 (5.56%)	5 (6.94%)	1.000
Other immunosuppressed state, n (%)	2 (5.41%)	2 (5.13%)	1.000	2 (5.41%)	2 (5.13%)	1.000	5 (9.26%)	2 (2.78%)	0.129
**Baseline SOFA scores**									
Cardiovascular, mean (SD)	1.41 (1.26)	1.23 (1.40)	0.220	3.27 (0.86)	3.48 (0.88)	0.061	3.65 (0.72)	3.72 (0.67)	0.286
Central nervous system, mean (SD)	3.41 (1.03)	3.28 (1.22)	0.358	3.71 (0.45)	3.73 (0.47)	0.342	3.94 (0.23)	3.93 (0.25)	0.379
Coagulation, mean (SD)	0.05 (0.32)	0.18 (0.45)	0.033	0.05 (0.22)	0.04 (0.20)	0.415	0.31 (0.74)	0.25 (0.55)	0.499
Liver, mean (SD)	0.27 (0.50)	0.13 (0.40)	0.059	0.17 (0.44)	0.12 (0.43)	0.148	0.37 (0.75)	0.36 (0.61)	0.369
Renal, mean (SD)	0.24 (0.67)	0.08 (0.35)	0.103	0.46 (0.63)	0.29 (0.69)	0.023	1.94 (1.45)	1.97 (1.44)	0.466
Respiration, mean (SD)	3.68 (0.70)	3.23 (1.00)	0.021	3.85 (0.52)	3.91 (0.41)	0.330	3.93 (0.38)	4.00 (0.00)	0.052
SOFA score, mean (SD)	9.05 (1.45)	8.13 (2.04)	0.009	11.51 (0.50)	11.57 (0.61)	0.164	14.15 (1.57)	14.24 (1.37)	0.253

*BMI* Body mass index, *HIV* human immunodeficiency virus, *NYP-WCMC* New York Presbyterian Hospital-Weill Cornell Medical Center, *SD* standard deviation, *SOFA* Sequential Organ Failure Assessment.

^†^p-value calculated by Chi-square test/Fisher’s exact test, or student’s t-test/Mann–Whitney test where appropriate.

** False discovery rate corrected p-value < 0.05.

### 30-Day clinical outcomes

**Figure 3:**
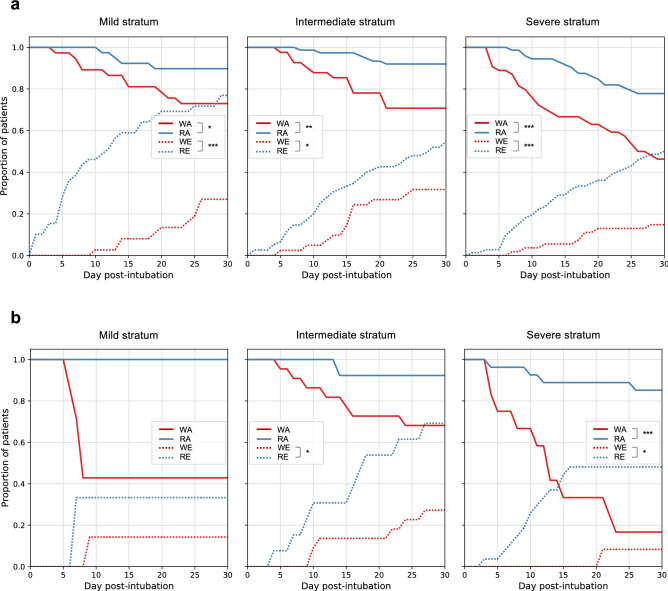
30-Day outcomes (extubation, mortality, and tracheostomy) of the trajectory subphenotypes. (**a**) 30-day outcomes of subphenotypes derived in NYP-WCMC cohort. (**b**) 30-day outcomes of subphenotypes derived in NYP-LMH validation cohort. Chi-square/Fisher’s exact tests were applied to compare 30-day outcomes between the worsening and recovering subphenotypes for each baseline strata. *Denoting testing significance passed p-value < 0.05; **denoting testing significance passed p-value < 0.01; ***denoting testing significance passed p-value < 0.001. *WA* worsening subphenotype alive, *RA* recovering subphenotype alive, *WE* worsening subphenotype extubated, *RE* recovering subphenotype extubated.

Statistics of 30-day post-intubation clinical primary and secondary outcomes (mortality, extubation, and tracheostomy) of subphenotypes were illustrated in Fig. 3a and Supplementary Fig. S3a. The worsening subphenotypes, across baseline strata, suffered from a significantly higher risk of mortality within the 30-day window after intubation (worsening vs recovering, mortality proportion: mild stratum, 29.7% vs. 10.3%, p = 0.033; intermediate stratum, 29.3% vs. 8.0%, p = 0.002; severe stratum, 53.7% vs. 22.2%, p < 0.001). The recovering subphenotypes, across all baseline strata, showed significantly higher extubation proportions within the 30-day window compared to the worsening subphenotypes (recovering vs. worsening, extubation proportion: mild stratum, 76.9% vs. 27.0%, p < 0.001; intermediate stratum, 54.7% vs. 31.7%, p = 0.018; severe stratum 50.0% vs. 14.8%, p < 0.001). There was no significant difference of 30-day tracheostomy detected between the subphenotypes. Importantly, the recovering subphenotype within the severe baseline stratum had a lower mortality risk compared to the worsening subphenotypes at mild and intermediate baseline strata.

The trajectory subphenotypes derived in the NYP-LMH validation cohort had similar trends in all three clinical outcomes within the 30-day window after intubation (see Fig. 3a and Supplementary Fig. S3b). Across all baseline strata, the worsening subphenotypes accounted for higher risk of mortality (worsening vs recovering, mortality proportion: mild stratum, 57.1% vs. 0.0%, p = 0.200; intermediate stratum, 31.8% vs. 7.7%, p = 0.211; severe stratum, 83.3% vs. 17.4%, p < 0.001), while the recovering subphenotypes showed higher extubation proportion within 30-days after intubation (recovering vs. worsening, extubation proportion: mild stratum, 33.3% vs. 14.3%, p = 0.490; intermediate stratum, 69.2% vs. 27.3%, p = 0.015; severe stratum, 48.1% vs. 9.1%, p = 0.017).

### Biomarkers of the trajectory subphenotypes

Vital signs, laboratory variables, and respiratory variables were first evaluated at baseline among the baseline strata. The three baseline strata of the NYP-WCMC cohort were observed to be well separated by a series of clinical variables in addition to the differential organ dysfunction pattern noted above (Supplementary Table S5). For instance, the severe strata had increased laboratory values like procalcitonin, ferritin, lactate dehydrogenase (LDH), and creatinine, and decreased bicarbonate at baseline. Additionally, vitals such as Glasgow Coma Scale (GCS), urine output volume and peak inspiratory pressure (PIP) were different across strata. Detailed statistical analyses are described in Supplementary Table S5. Statistics of these clinical variables across baseline strata within the NYP-LMH validation cohort showed similar signals and were detailed in Supplementary Table S6.

**Figure 4:**
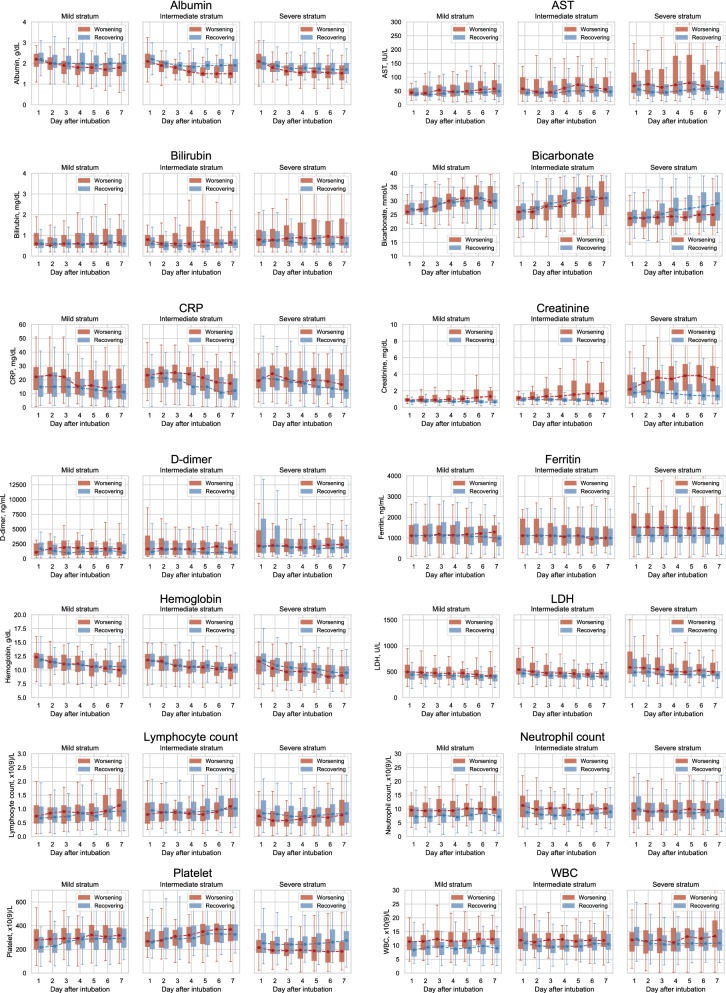
Laboratory test value trajectories of the identified subphenotypes. *AST* Aspartate aminotransferase, *CK* creatine kinase, *CRP* C-reactive protein, *LDH* lactate dehydrogenase.

We further compared the 7-day post-intubation trajectories of the clinical variables and biomarkers between the worsening and recovering subphenotypes within each stratum (Fig. 4, Supplementary Figs. S4–S6, and Supplementary Table S7). Across the three baseline severity strata, the serum albumin had a lower nadir in the worsening compared to that of the recovering subphenotypes (Fig. 4). GCS recovery was associated with overall improvement (Supplementary Fig. S5). Additionally, the PaO_2_/FiO_2_ ratio (P/F ratio) was lower within the 7-day window (Supplementary Fig. S6). Moreover, positive end-expiratory pressure (PEEP) and PIP improved within the recovering subphenotypes, while it failed to improve within the worsening subphenotypes (Supplementary Fig. S6).

Within the mild stratum, general inflammatory markers such as C-reactive protein (CRP), white blood cell (WBC) count, and neutrophil count were higher at baseline and remained higher within the 7-day window after intubation, compared to those of the recovering subphenotype (Fig. 4, Supplementary Table S8). Additionally, despite a similar baseline, mean arterial pressure (MAP) increased within the recovering subphenotype compared to the worsening subphenotype (Supplementary Fig. S5 and Supplementary Table S8). The intermediate stratum also had higher sustained general inflammatory markers in the worsening subphenotype compared to the improving (Fig. 4, Supplementary Table S8).

In the severe stratum general inflammatory markers were similar in the worsening and improving subphenotypes. However, there was higher aspartate aminotransferase (AST), ferritin, LDH, creatine kinase (CK), procalcitonin, and troponin in the worsening subphenotype compared to the improving. The worsening subphenotype had increasing serum bilirubin, creatinine and falling platelets and hemoglobin compared to the recovering subphenotype (Fig. 4). In addition, the platelet and urine output fell in the worsening subphenotype, while those within the recovering subphenotype had a clear improvement (Fig. 4, Supplementary Fig. S5).

Most markers identified within the NYP-WCMC cohort showed consistent signals within the NYP-LMH subphenotypes (Supplementary Table S8).

### Subphenotype prediction models

We trained random forest models for predicting the worsening and recovering trajectory subphenotypes within each baseline stratum according to the early stage marker values. Overall, as shown in Supplementary Fig. S7, within the mild, intermediate, and severe strata, the prediction models achieved the AUC-ROCs of 0.71 (95% confidence interval [CI] [0.67, 0.78]), 0.69 (95% CI [0.67, 0.71]), and 0.74 (95% CI [0.71, 0.77]) respectively, with the predictor values evaluated at day 1 post-intubation. AUC-ROCs of the models increased to 0.78 (95% CI [0.72, 0.84]), 0.77 (95% CI [0.76, 0.79]), and 0.79 (95% CI [0.77, 0.82]), with the predictor values evaluated at day 3 post-intubation; and to 0.83 (95% CI [0.79, 0.88]), 0.91 (95% CI [0.89, 0.93]), and 0.88 (95% CI [0.84, 0.92]), with the predictor values evaluated at day 5 post-intubation. Similar patterns of prediction performances in terms of AUC-PR scores were observed as well (Supplementary Fig. S7).

Importance of the predictors were illustrated as heatmaps, where color intensity represents the normalized importance of specific predictors (Supplementary Fig. S8). Generally, predictor importance varied as the progress of time. Models trained on day 1–3 after intubation were observed to involve more contributions from the laboratory tests, vital signs, respiratory variables than other predictors; SOFA subscores, especially cardiovascular, CNS, and renal subscores showed relatively higher importance over models trained on day 4 or 5 data within the intermediate and severe strata. Age contributed to day 1–3 prediction to some extent, while other demographics, medications and comorbidities showed weak importance in prediction.

## Discussion

In this study, we identified novel trajectory subphenotypes of COVID-19 patients with an objective machine learning approach. The subphenotypes we identified are based on organ dysfunction trajectory over 7-days following intubation, which is different from existing data-driven subphenotyping methods that focus on patient data at a specific timestamp^15,35,36^. The use of novel methodology, in addition to the robust size of our cohort, ensure that the identified trajectory based subphenotypes are less likely to suffer from cognitive bias^16^ and are likely to be temporally stable^37^. More concretely, we adopted a divide and conquer approach to identify the subphenotypes. Prior research has identified that additive organ dysfunction is predictive of increased mortality in COVID-19 associated ARDS^11^. Therefore, we divided the patients into three different baseline strata (mild, intermediate and severe) according to additive SOFA based organ dysfunction. Patients within each stratum had homogenous organ dysfunctions at baseline. We identified two salient trajectory subphenotypes within each stratum, aligned at the time of intubation.

Importantly, the baseline demographics, comorbidities and pattern of organ dysfunction did not differ between the worsening and recovering subphenotypes at each stratum. This suggests the existence of differential progression pathways that are irrespective of baseline risk factors for severe disease. This finding is unique compared to other subphenotyping projects as we are including a more complete picture of the disease course^15,35,36^. It also highlights the temporal heterogeneity of COVID-19 and the importance of avoiding prognostication based on early post intubation clinical characteristics. We found that the worsening subphenotypes in the baseline mild and intermediate strata showed an even higher risk of death compared to the recovering subphenotype within the baseline severe stratum (Fig. 3). Indeed, there is an ongoing need to understand the pathophysiology of progressive non-pulmonary organ dysfunction in this disease.

We assessed the differences between a broad range of laboratory tests, vital signs, and respiratory variables in the worsening and recovering subphenotypes. Importantly, 7-day trajectories of these variables showed that different markers contributed to separating the worsening and recovering subphenotypes across different strata. Specifically, inflammatory markers such as CRP, neutrophil count, and WBC differentiated worsening and recovering trends within the mild strata (Fig. 4). In contrast to the mild stratum, higher ferritin, increasing bilirubin, LDH, and creatinine, as well as decreasing platelets and hemoglobin suggest that worsening within the severe baseline stratum is driven by cell death, macrophage activation and overt organ dysfunction with disseminated intravascular coagulation^38^. These observations suggest differential underlying mechanisms of the worsening and recovering subphenotypes across baseline severity strata. In this context, the novel subphenotypes could be incorporated in future randomized clinical trials. The biomarker profiles also suggest potential overlaps in biological mechanisms between our identified subphenotypes with those in the traditional ARDS population^15^. Especially, the increasing creatinine and decreasing albumin, platelet count, and bicarbonate of the worsening subphenotype within the severe baseline stratum showed that it seems analogous to the hyperinflammatory subphenotype in the non-COVID ARDS population.

We built multivariable prediction models for the identified trajectory subphenotypes from patient baseline characteristics and early-stage clinical feature values. Models were built on at successive time points (day 1, 2, 3, 4, and 5) after intubation. Predictive performances measured by AUC-ROC improved as the number of days increased. The predictors’ importance to differentiating worsening and recovering subphenotypes showed varying patterns that were similar to differences over time described above (Fig. 4, Supplementary Fig. S8). Importantly, aside from age and BMI, demographics, baseline comorbidities, and medications prescribed around intubation did not contribute to discriminating the subphenotypes in any of the strata.

Our study was conducted on the two NYP system hospitals. Woresning and recovering SOFA subphenotypes, clinical characteristics, and outcomes from the validation cohort was consistent with the original subphenotypes. Although, due to the limited size of NYP-LMH validation cohort, statistical significance of some markers vanished, most of the results reflected the development cohort’s findings. This consistency ensures the existence of the worsening and recovering trajectory subphenotypes at each baseline stratum of the critically ill COVID-19 patients.

While this study presents a step forward in the efforts to parse the progression heterogeneity of critically ill patients with COVID-19, several limitations remain. The first limitation could be SOFA’s inadequacy in tabulating organ dysfunction in COVID-19 associated respiratory failure^39^. Despite this potential limitation, SOFA trajectory subphenotypes predicted mortality and importantly will allow for comparisons with other diseases in the future. Additionally, our analysis was aligned at the time of intubation to capture patients at a similar point in their disease course. However, it is known that there is significant variation in the timing of intubation between institutions and providers in the setting of acute respiratory failure^40,41^. It is possible that our observed progression patterns may be confounded by patients being intubated at different points in their disease.

Second, we did not build our subphenotypes with inflammatory markers such as C-reactive protein, d-dimer or ferritin, which are known risk factors for this disease. Instead, we chose to explore how these factors interact with traditional organ dysfunction as this is more closely related to mortality. Nor did we stratify patients based the severity of respiratory failure alone. Instead, we chose to see how respiratory failure interacted with organ dysfunction, as most patients with COVID19 die from multisystem organ failure^11,12^.

Third, differentiating trajectory subphenotypes in this critically ill population was difficult, as AUC-ROC metrics of prediction modeling using data at day 1 post-intubation were around 0.7. By restricting our analysis to a very high-risk population, we decreased the discriminative power of many of our biomarkers to predict outcomes. All patients were high risk. However, we have documented the natural history of organ dysfunction in critical COVID-19 and explored the interaction between organ failure and clinical inflammatory biomarkers. Future research efforts, with novel biomarkers, are needed to predict worsening and recovering subphenotypes at an earlier time point in critical COVID-19.

Fourth, the surge conditions in New York City during the study period could affect the study. Care may have been influenced by the surge conditions during this difficult time. However, all patients were cared for in a critical care environment and despite the massive patient burden, the all cause 30-day mortality was 25.9%.

Fifth, though the data-driven methods are free from cognitive biases of the subphenotypes^16^, our analysis may account for cognitive bias. For instance, the progression to multi-organ failure could be synonymous with death, and hence results in cognitive traps.

## Conclusions

In a population of critically ill patients with COVID-19 respiratory failure, there are distinct worsening and recovering organ dysfunction trajectory subphenotypes. Worsening status was predictive of poor outcomes in all strata regardless of baseline severity and was associated with different patterns of biomarker alteration. These findings highlight the importance stratification within critical COVID-19 when evaluating potential therapies. Trajectory based subphenotypes offer a road map for understanding the evolution of critical illness in COVID-19. We call for further analysis.

## Supplementary Information

Supplementary Information.

## Data Availability

The raw dataset generated or analyzed during this study is not publicly available due to the patient privacy/consent.
