# Supplementary material for: Identifying organ dysfunction trajectory-based subphenotypes in critically ill patients with COVID-19
Source: Sci Rep. 2021 Aug 5;11:15872. doi: 10.1038/s41598-021-95431-7 (PMC8342520; doi:10.1038/s41598-021-95431-7)
Supplement: Supplementary file 1 — Supplementary Information. [file 41598_2021_95431_MOESM1_ESM.pdf]

Supplementary online resource

Identifying organ dysfunction trajectory-based subphenotypes in critically ill patients with COVID-19

Authors: Chang Su, PhD[1], Zhenxing Xu, PhD[1], Katherine Hoffman, MS[1], Parag Goyal, MD[2,3], Monika M Safford, MD[2,3], Jerry Lee, BA[4], Sergio Alvarez-Mulett MD[3,5], Luis Gomez-Escobar, MD[3,5], David R Price, MD[3,5], John S Harrington, MD[3,5], Lisa K Torres, MD[3,5], Fernando J Martinez, MD[3,5], Thomas R. Campion, Jr., PhD[1], Fei Wang, PhD[1], Edward J. Schenck, MD[3,5]

[1]Department of Population Health Sciences, Weill Cornell Medicine, New York, NY, USA;

[2]Division of General Internal Medicine, Joan and Sanford I. Weill Department of Medicine, Weill Cornell Medicine, New York, NY, USA;

[3]New York-Presbyterian Hospital, Weill Cornell Medicine, New York, NY, USA;

[4]Weill Cornell Medical College, Weill Cornell Medicine, New York, NY, USA;

[5]Division of Pulmonary & Critical Care Medicine, Joan and Sanford I. Weill Department of Medicine, Weill Cornell Medicine, New York, NY, USA.

Corresponding authors:

Fei Wang, few2001@med.cornell.edu

Edward J. Schenck, ejs9005@med.cornell.edu

Appendix 1. Patient care.

Care of the patient was at the discretion of each attending physician. Patients were intubated prior to arrival to the ICU. Daily briefings were held during the surge to reinforce best practices and evidence related to caring for acute hypoxemic respiratory failure. Volume control ventilation targeting a tidal volume of 6-8 mls/kg IDBW and a plateau pressure < 30 cm/h20 was recommended. No particular PEEP titration protocol was mandated, however the ARDSnet moderate and high PEEP documents were made available to practitioners. Propofol was the first sedative of choice with the addition of opiates for dyspnea and perceived pain. An even to positive fluid balance was suggested on days one and two of mechanical ventilation. Patients with hypotension despite volume loading were treated with vasopressors, norepinephrine as the first choice to target a MAP of 65 mmhg. Prone positioning was suggested if a patient had a PF ratio of <150 despite optimization of PEEP and vent synchrony. Neuromuscular blockade was suggested in cases of refractory hypoxemia. Inhaled nitric oxide was available in cases of refractory respiratory failure at the discretion of the treating intensivists. Extra corporeal membrane oxygenation support was not offered due to resource limitations. HProphylactic anticoagulation was recommended for all patients and a higher dose prophylactic regimen was implemented during the course of the surge. Enoxaparin was the recommended first choice anticoagulation, even in the setting of renal insufficiency with strict monitoring of anti-factor Xa levels. Heparin was used for patients with concern for active bleeding or patients unable to tolerate enoxaparin. Corticosteroids were used at the discretion of the individual attending physician for the treatment of ARDS and for other traditional complications such as septic shock, bronchospasm and airway edema. Hydroxychloroquine without azithromycin was used to treat most patients as an antiviral. Remdesivir was available through compassionate use and through several clinical trials. Off label use of IL-6 inhibition was directed by consultation with infectious

disease physicians. Choices regarding antibiotics and other therapeutics were at the discretion of treating intensivists with consultation with infectious disease physicians.

Appendix 2. Data description.

In this study, all data were collected from either the Weill Cornell-Critical carE Database for Advanced Research (WC-CEDAR)[1], Weill Cornell Medicine COVID Institutional Data Repository (COVID-IDR)[2], or via manual chart abstraction (REDCap). A wide range of data were included for analysis, including:

- **Demographics**. Demographics include age, sex, race, and body mass index (BMI) were obtained when admitted to ICU.
- **Comorbidities**. Chronic comorbidities were assessed at ICU admission and collected via chart abstraction. Comorbidities studied include coronary artery disease, cerebrovascular accident (stroke), heart failure, hypertension, diabetes mellitus, pulmonary disease, renal disease, cirrhosis, hepatitis, active cancer, transplant, inflammatory bowel disease, human immunodeficiency viruses, rheumatologic disease, and other immunosuppressed state.
- **Medications**. Medications prescribed were collected from the COVID-IDR database. Medications screened include tocilizumab, hydroxychloroquine, corticosteroids (such as prednisone, methylprednisolone, dexamethasone, and hydrocortisone), enoxaparin, heparin, and antibiotics (such as ceftriaxone, azithromycin, piperacillin-tazobactam, meropenem, vancomycin, and doxycycline). Enoxaparin was further assessed as prophylactic dose (<.5 mg per KG), high prophylactic dose (<1 mg per KG), and treatment dose (>1 mg per KG), while heparin

was assessed as prophylactic dose (subcutaneous delivery) and treatment dose (intravenous delivery).

- **Laboratory test values**. In order to identify markers associated to identified subphenotypes, we assessed a broad spectrum of laboratory tests related to organ failure, including alanine aminotransferase (ALT), albumin level, aspartate aminotransferase (AST), total bilirubin, bicarbonate, creatine kinase (CK) level, creatinine, C-reactive protein (CRP) level, D-dimer, erythrocyte sedimentation rate (ESR), ferritin level, globulin level, glucose level, hemoglobin level, lactate dehydrogenase (LDH), lactic acid level, lymphocyte percentage and count, neutrophil percentage and count, platelets level, potassium level, procalcitonin level, sodium level, troponin level, triglycerides, and white blood cell (WBC) count.
- **Vital signs**. Several vital signs were also assessed, including Glasgow Coma Scale score (GCS), and mean arterial pressure (MAP), temperature.
- **Respiratory variables**. Variables reflecting respiratory status of patients were also studied, including fraction of inspired oxygen (FiO2), partial pressure of oxygen (PaO2), PaO2/FiO2 ratio (P/F ratio), and oxygen saturation (SpO2). Several parameters of ventilator setting were studied, including driving pressure, minute ventilation, arterial partial pressure of carbon dioxide (PCO2) level, positive end-expiratory pressure (PEEP), PH, peak inspiratory pressure (PIP), plateau pressure, static compliance, tidal volume, tidal volume to predicted body weight (PBW) ratio, and ventilator ratio.

Clinical variables including laboratory test values, vital signs, and respiratory variables were collected daily. If more than one result was recorded on a given day, the average value was selected at data entry.

Appendix 3. Inclusion exclusion criteria

Inclusion: We included patients with positive results on viral RNA detection by real-time reverse transcriptase polymerase chain reaction (RT-PCR) test from nasopharyngeal swabs specimens and treated with mechanical ventilation at the ICU in NYP-WCMC and NYP-LMH.

Exclusion:

- Patients who were less than 18 years old when admitted to ICU were excluded for analysis.
- Since our aim was to identify organ dysfunction progression patterns within 7-days after intubation, missing too many (>50%) SOFA data may mislead understanding the trajectory trends. Hence, we excluded patients with ≤3 days SOFA data. Specifically, 20 patients of NYP-WCMC cohort were excluded, among which 9, 3, and 3 dead within day 1, 2, 3 after intubation, respectively, 2 have no records, and 2 and 1 only have 2- and 3-days SOFA data, respectively. 11 patients of NYP-LMH cohort were excluded, among which 2, 5, and 4 dead within day 1, 2, 3 after intubation, respectively.
- Outliers such as unchanged or heavily fluctuated trajectories which may hamper the model to capture clinically meaningful SOFA progression trends were excluded for analysis. Specifically, 7 unchanged trajectories of NYP-WCM cohort, and 3 and 5 heavily fluctuated trajectories within 7-day post-intubation of NYP-WCMC and NYP-LMH cohorts were excluded for analysis.

The overall inclusion exclusion criteria were provided in Figure S1.

References

1. Sholle, E. T. *et al.* Secondary Use of Patients' Electronic Records (SUPER): An Approach for Meeting Specific Data Needs of Clinical and Translational Researchers. *AMIA Annu Symp Proc* **2017**, 1581-1588 (2017).
2. Goyal, P. *et al.* Clinical Characteristics of Covid-19 in New York City. *N. Engl. J. Med.* **382**, 2372-2374, doi:10.1056/NEJMc2010419 (2020).
3. McClain, J. O. & Rao, V. R. CLUSTISZ: A Program to Test for the Quality of Clustering of a Set of Objects. *J. Mark. Res.* **12**, 456-460 (1975).

Tables

Table S1. Clustering metric (McClain index[3]) statistics and cluster number determination.

	NYP-WCMC cohort			NYP-LMH validation cohort		
**# of clusters**	**Mild stratum**	**Intermediate stratum**	**Severe stratum**	**Mild stratum**	**Intermediate stratum**	**Severe stratum**
2	0.53	0.50	0.54	0.28	0.59	0.48
3	0.60	0.58	0.70	0.32	0.66	0.59
4	0.88	1.05	0.74	0.34	1.13	0.73
5	0.88	1.10	0.76	0.49	1.23	1.04
6	1.15	1.12	0.86	0.72	1.66	1.11
**Optimal cluster #**	2	2	2	2	2	2

The minimum value of the McClain index is used to indicate the optimal number of clusters.

Table S2. Clinical characteristics of the trajectory subphenotypes in NYP-LMH validation cohort

	Mild stratum (SOFA 0-10, n=10)			Intermediate stratum (SOFA 11-12, n=35)			Severe stratum (SOFA 13-24, n=39)		
**Variable**	**Worsening**	**Recovering**	**p-value†**	**Worsening**	**Recovering**	**p-value†**	**Worsening**	**Recovering**	**p-value†**
**Total #**	**7**	**3**	**-**	**22**	**13**	**-**	**12**	**27**	**-**
Demographics									
Age, Mean (SD)	60.29 (19.72)	62.67 (7.93)	0.861	60.73 (12.40)	63.15 (9.47)	0.559	78.00 (9.08)	68.37 (10.58)	0.011
Sex female, n (%)	4 (57.14%)	0 (0.00%)	0.200	10 (45.45%)	9 (69.23%)	0.293	2 (16.67%)	8 (29.63%)	0.693
CAUCASIAN, n (%)	0 (0.00%)	0 (0.00%)		2 (9.09%)	2 (15.38%)		2 (16.67%)	1 (3.70%)	
AFRICAN AMERICAN, n (%)	0 (0.00%)	0 (0.00%)	1.000	0 (0.00%)	0 (0.00%)	0.149	3 (25.00%)	4 (14.81%)	0.345
ASIAN / PACIFIC ISLANDER, n (%)	3 (42.86%)	2 (66.67%)		9 (40.91%)	3 (23.08%)		3 (25.00%)	12 (44.44%)	
MULTI-RACIAL, n (%)	2 (28.57%)	0 (0.00%)		1 (4.55%)	4 (30.77%)		0 (0.00%)	3 (11.11%)	
BMI, Mean (SD)	26.77 (4.64)	26.43 (1.14)	0.914	31.22 (11.98)	28.12 (4.57)	0.262	26.90 (3.72)	28.58 (6.25)	0.409
Comorbidities									
Coronary Artery Disease, n (%)	1 (14.29%)	0 (0.00%)	1.000	0 (0.00%)	1 (7.69%)	0.323	4 (33.33%)	5 (18.52%)	0.416
Cerebrovascular accident (Stroke), n (%)	0 (0.00%)	0 (0.00%)	1.000	0 (0.00%)	0 (0.00%)	1.000	3 (25.00%)	1 (3.70%)	0.078
Heart Failure, n (%)	0 (0.00%)	0 (0.00%)	1.000	0 (0.00%)	1 (7.69%)	0.323	0 (0.00%)	2 (7.41%)	1.000
Hypertension, n (%)	3 (42.86%)	2 (66.67%)	1.000	9 (40.91%)	8 (61.54%)	0.068	7 (58.33%)	21 (77.78%)	0.262
Diabetes Mellitus, n (%)	3 (42.86%)	1 (33.33%)	1.000	7 (31.82%)	5 (38.46%)	0.447	5 (41.67%)	14 (51.85%)	0.731
Pulmonary Disease, n (%)	1 (14.29%)	1 (33.33%)	1.000	2 (9.09%)	2 (15.38%)	0.577	3 (25.00%)	6 (22.22%)	1.000
Renal Disease, n (%)	0 (0.00%)	0 (0.00%)	1.000	0 (0.00%)	2 (15.38%)	0.097	0 (0.00%)	5 (18.52%)	0.299
Cirrhosis, n (%)	0 (0.00%)	0 (0.00%)	1.000	0 (0.00%)	0 (0.00%)	1.000	0 (0.00%)	1 (3.70%)	1.000
Hepatitis, n (%)	0 (0.00%)	0 (0.00%)	1.000	1 (4.55%)	0 (0.00%)	1.000	0 (0.00%)	1 (3.70%)	1.000
HIV, n (%)	0 (0.00%)	0 (0.00%)	1.000	1 (4.55%)	0 (0.00%)	1.000	0 (0.00%)	0 (0.00%)	1.000
Active Cancer, n (%)	0 (0.00%)	0 (0.00%)	1.000	0 (0.00%)	0 (0.00%)	1.000	1 (8.33%)	1 (3.70%)	0.526
Transplant, n (%)	0 (0.00%)	0 (0.00%)	1.000	0 (0.00%)	0 (0.00%)	1.000	0 (0.00%)	1 (3.70%)	1.000
Inflammatory Bowel Disease, n (%)	0 (0.00%)	0 (0.00%)	1.000	0 (0.00%)	0 (0.00%)	1.000	0 (0.00%)	0 (0.00%)	1.000
Rheumatologic Disease, n (%)	0 (0.00%)	0 (0.00%)	1.000	0 (0.00%)	2 (15.38%)	0.097	1 (8.33%)	0 (0.00%)	0.308
Other Immunosuppressed State, n (%)	0 (0.00%)	0 (0.00%)	1.000	0 (0.00%)	0 (0.00%)	1.000	0 (0.00%)	0 (0.00%)	1.000
Baseline SOFA scores									
Cardiovascular, Mean (SD)	1.43 (1.05)	1.33 (0.94)	0.500	3.50 (0.78)	3.69 (0.82)	0.169	4.00 (0.00)	3.81 (0.47)	0.087

Central nervous system, Mean (SD)	2.29 (1.48)	3.33 (0.47)	0.183	3.27 (0.45)	3.54 (0.50)	0.063	3.42 (0.49)	3.70 (0.53)	0.039
Coagulation, Mean (SD)	0.00 (0.00)	0.00 (0.00)	-	0.18 (0.49)	0.00 (0.00)	0.091	0.33 (0.62)	0.11 (0.31)	0.127
Liver, Mean (SD)	0.14 (0.35)	0.00 (0.00)	0.331	0.23 (0.52)	0.00 (0.00)	0.057	0.42 (0.64)	0.22 (0.50)	0.160
Renal, Mean (SD)	0.43 (0.73)	0.67 (0.47)	0.257	0.36 (0.48)	0.38 (0.84)	0.270	2.33 (0.85)	2.52 (1.17)	0.307
Respiration, Mean (SD)	4.00 (0.00)	4.00 (0.00)	-	3.91 (0.42)	4.00 (0.00)	0.239	4.00 (0.00)	4.00 (0.00)	-
SOFA score, Mean (SD)	8.29 (2.43)	9.33 (0.47)	0.500	11.45 (0.50)	11.62 (0.49)	0.188	14.50 (1.04)	14.37 (1.09)	0.377

† p-value calculated by Chi-square test/Fisher's exact test, or student's t-test/Mann-Whitney test where appropriate.

** False discovery rate corrected p-value < 0.05

Abbreviation: BMI=body mass index, HIV=Human Immunodeficiency Virus, NYP-LMH=New York Presbyterian Hospital-Lower Manhattan Hospital, SD=standard deviation, SOFA=Sequential Organ Failure Assessment

Table S3. Medication of the trajectory subphenotypes in NYP-WCMC cohort

Medication	Mild stratum (SOFA 0-10, n=76)			Intermediate stratum (SOFA 11-12, n=116)			Severe stratum (SOFA 13-24, n=126)		
	Worsening (n=39)	Recovering (n=37)	p-value†	Worsening (n=41)	Recovering (n=75)	p-value†	Worsening (n=54)	Recovering (n=72)	p-value†
Tocilizumab, n (%)	4 (10.81%)	3 (7.69%)	0.708	5 (12.20%)	5 (6.67%)	0.321	4 (7.41%)	4 (5.56%)	0.724
Hydroxychloroquine, n (%)	29 (78.38%)	27 (69.23%)	0.439	35 (85.37%)	51 (68.00%)	0.048	41 (75.93%)	52 (72.22%)	0.686
Prednisone, n (%)	0 (0.00%)	4 (10.26%)	0.116	1 (2.44%)	1 (1.33%)	1.000	3 (5.56%)	2 (2.78%)	0.651
Methylprednisolone, n (%)	7 (18.92%)	10 (25.64%)	0.586	8 (19.51%)	16 (21.33%)	1.000	11 (20.37%)	8 (11.11%)	0.208
Dexamethasone, n (%)	1 (2.70%)	2 (5.13%)	1.000	1 (2.44%)	4 (5.33%)	0.655	0 (0.00%)	2 (2.78%)	0.506
Hydrocortisone, n (%)	5 (13.51%)	1 (2.56%)	0.103	7 (17.07%)	6 (8.00%)	0.216	15 (27.78%)	8 (11.11%)	0.020
Ceftriaxone, n (%)	18 (48.65%)	21 (53.85%)	0.819	23 (56.10%)	43 (57.33%)	1.000	25 (46.30%)	40 (55.56%)	0.369
Azithromycin, n (%)	8 (21.62%)	7 (17.95%)	0.777	5 (12.20%)	13 (17.33%)	0.595	8 (14.81%)	17 (23.61%)	0.264
Piperacillin-tazobactam, n (%)	12 (32.43%)	13 (33.33%)	1.000	25 (60.98%)	29 (38.67%)	0.032	24 (44.44%)	36 (50.00%)	0.591
Meropenem, n (%)	4 (10.81%)	4 (10.26%)	1.000	3 (7.32%)	3 (4.00%)	0.664	12 (22.22%)	7 (9.72%)	0.077
Vancomycin, n (%)	10 (27.03%)	12 (30.77%)	0.803	25 (60.98%)	23 (30.67%)	0.003	24 (44.44%)	32 (44.44%)	1.000
Doxycycline, n (%)	19 (51.35%)	22 (56.41%)	0.818	19 (46.34%)	34 (45.33%)	1.000	21 (38.89%)	34 (47.22%)	0.370
Enoxaparin, n (%)									
Prophylactic dose (<.5 mg/Kg)	8 (21.62%)	10 (25.64%)	0.117	9 (21.95%)	15 (20.00%)	0.824	14 (25.93%)	17 (23.61%)	0.241
High prophylactic dose (>.5 mg/Kg)	13 (35.14%)	21 (53.85%)		17 (41.46%)	37 (49.33%)		10 (18.52%)	28 (38.89%)	
Treatment dose (>1 mg/Kg)	8 (21.62%)	6 (15.38%)		8 (19.51%)	14 (18.67%)		7 (12.96%)	7 (9.72%)	
Heparin, n (%)									
Prophylactic dose (subcutaneous)	5 (13.51%)	9 (23.08%)	0.539	13 (31.71%)	9 (12.00%)	<0.001**	18 (33.33%)	25 (34.72%)	0.170
treatment dose	3 (8.11%)	3 (7.69%)		8 (19.51%)	2 (2.67%)		18 (33.33%)	14 (19.44%)	

Medications were prescribed within the window from 3-day before to 5-day after intubation.
† p-value calculated by Chi-square/Fisher's exact test
** False discovery rate corrected p-value < 0.05
Abbreviation: BMI=body mass index, HIV=Human Immunodeficiency Virus, NYP-WCMC=New York Presbyterian Hospital-Weill Cornell Medical Center, SD=standard deviation, SOFA=Sequential Organ Failure Assessment

Table S4. Medication of the trajectory subphenotypes in NYP-LMH cohort

Medication	Mild stratum (SOFA 0-10, n=10)			Intermediate stratum (SOFA 11-12, n=35)			Severe stratum (SOFA 13-24, n=39)		
	Worsening (n=7)	Recovering (n=3)	p-value†	Worsening (n=22)	Recovering (n=13)	p-value†	Worsening (n=12)	Recovering (n=27)	p-value†
Tocilizumab, n (%)	0 (0.00%)	0 (0.00%)	1.000	4 (18.18%)	1 (7.69%)	0.635	1 (8.33%)	5 (18.52%)	0.645
Hydroxychloroquine, n (%)	6 (85.71%)	3 (100.00%)	1.000	19 (86.36%)	11 (84.62%)	1.000	10 (83.33%)	22 (81.48%)	1.000
Prednisone, n (%)	0 (0.00%)	0 (0.00%)	1.000	0 (0.00%)	0 (0.00%)	1.000	0 (0.00%)	1 (3.70%)	1.000
Methylprednisolone, n (%)	2 (28.57%)	2 (66.67%)	0.500	8 (36.36%)	3 (23.08%)	0.705	2 (16.67%)	7 (25.93%)	0.693
Dexamethasone, n (%)	0 (0.00%)	1 (33.33%)	0.300	0 (0.00%)	0 (0.00%)	1.000	0 (0.00%)	0 (0.00%)	1.000
Hydrocortisone, n (%)	1 (14.29%)	0 (0.00%)	1.000	0 (0.00%)	1 (7.69%)	0.353	5 (41.67%)	6 (22.22%)	0.262
Ceftriaxone, n (%)	3 (42.86%)	3 (100.00%)	0.200	17 (77.27%)	9 (69.23%)	1.000	6 (50.00%)	15 (55.56%)	1.000
Azithromycin, n (%)	4 (57.14%)	1 (33.33%)	1.000	11 (50.00%)	4 (30.77%)	0.476	4 (33.33%)	8 (29.63%)	1.000
Piperacillin-tazobactam, n (%)	5 (71.43%)	1 (33.33%)	0.500	11 (50.00%)	8 (61.54%)	0.476	8 (66.67%)	14 (51.85%)	0.494
Meropenem, n (%)	0 (0.00%)	0 (0.00%)	1.000	0 (0.00%)	1 (7.69%)	0.353	0 (0.00%)	0 (0.00%)	1.000
Vancomycin, n (%)	3 (42.86%)	1 (33.33%)	1.000	9 (40.91%)	8 (61.54%)	0.282	10 (83.33%)	15 (55.56%)	0.151
Doxycycline, n (%)	2 (28.57%)	2 (66.67%)	0.500	10 (45.45%)	6 (46.15%)	1.000	7 (58.33%)	11 (40.74%)	0.488
Enoxaparin, n (%)									
Prophylactic dose (<.5 mg/Kg)	2 (28.57%)	0 (0.00%)		10 (45.45%)	5 (38.46%)		3 (25.00%)	8 (29.63%)	
High prophylactic dose (>.5 mg/Kg)	4 (57.14%)	2 (66.67%)	1.000	8 (36.36%)	7 (53.85%)	0.446	1 (8.33%)	10 (37.04%)	0.075
Treatment dose (>1 mg/Kg)	0 (0.00%)	0 (0.00%)		1 (4.55%)	0 (0.00%)		1 (8.33%)	4 (14.81%)	
Heparin, n (%)									
Prophylactic dose (subcutaneous)	4 (57.14%)	1 (33.33%)	1.000	12 (54.55%)	5 (38.46%)	0.205	7 (58.33%)	11 (40.74%)	0.503
treatment dose	0 (0.00%)	0 (0.00%)		3 (13.64%)	0 (0.00%)		3 (25.00%)	7 (25.93%)	

Medications were prescribed within the window from 3-day before to 5-day after intubation.
† p-value calculated by Chi-square/Fisher's exact test
** False discovery rate corrected p-value < 0.05
Abbreviation: BMI=body mass index, HIV=Human Immunodeficiency Virus, NYP-LMH=New York Presbyterian Hospital-Lower Manhattan Hospital, SD=standard deviation, SOFA=Sequential Organ Failure Assessment

Table S5. Clinical variables (laboratory test results, vital signs, respiratory variables) of the baseline strata in NYP-WCMC cohort. Data were examined at day 1 post-intubation.

Variable	All	Mild stratum	Intermediate stratum	Severe stratum	p-value†	Post-hoc
ALT, IU/L, Median [IQR]	42.0 [26.0-70.75]	39.0 [23.75-59.12]	42.0 [27.38-70.25]	44.5 [26.25-74.0]	0.631	
Albumin, g/dL, Median [IQR]	2.1 [1.8-2.49]	2.2 [1.9-2.5]	2.2 [1.94-2.4]	2.0 [1.7-2.45]	0.109	
AST, IU/L, Median [IQR]	49.0 [35.0-79.5]	40.5 [33.5-61.0]	48.0 [34.75-65.75]	59.75 [35.0-98.0]	0.005**,§	Severe vs Mild, Intermediate
Bilirubin, mg/dL, Median [IQR]	0.7 [0.5-1.0]	0.6 [0.5-0.9]	0.7 [0.47-0.9]	0.78 [0.5-1.2]	0.247	
Bicarbonate, mmol/L, Median [IQR]	25.42 [23.0-28.0]	26.75 [25.0-29.0]	26.0 [23.25-28.62]	24.0 [21.75-26.38]	<0.001**,§	Severe vs Mild, Intermediate
CK, U/L, Median [IQR]	181.0 [77.0-326.75]	172.25 [61.75-253.5]	154.25 [79.75-274.63]	187.0 [77.25-407.75]	0.177	
Creatinine, mg/dL, Median [IQR]	1.1 [0.8-1.77]	0.81 [0.67-0.97]	0.98 [0.77-1.26]	1.86 [1.29-3.43]	<0.001**,§	All comparison
CRP, mg/dL, Median [IQR]	20.25 [12.95-27.9]	18.65 [10.8-26.23]	20.94 [13.57-27.52]	20.11 [13.6-28.08]	0.428	
D-dimer, ng/mL, Median [IQR]	1331.75 [734.0-3185.0]	1087.5 [596.75-2373.95]	1331.75 [692.38-3023.5]	1942.62 [1009.0-4408.14]	0.003**,§	Severe vs Mild, Intermediate
ESR, mm/hr, Median [IQR]	83.0 [67.25-97.38]	83.0 [72.92-100.25]	83.0 [67.0-89.06]	83.0 [66.58-100.0]	0.243	
Ferritin, ng/mL, Median [IQR]	1166.3 [772.52-1639.7]	1121.9 [601.9-1611.3]	1166.3 [636.66-1596.6]	1283.45 [927.74-1766.8]	0.014**,§	Severe vs Mild, Intermediate
Globulin, g/dL, Median [IQR]	3.4 [3.1-3.8]	3.3 [3.1-3.7]	3.4 [3.14-3.8]	3.4 [3.0-3.89]	0.503	
Glucose, mg/dL, Median [IQR]	142.5 [113.5-190.38]	118.25 [103.88-146.25]	145.5 [119.25-189.25]	154.0 [124.0-207.5]	<0.001**,§	Mild vs Intermediate, Severe
Hemoglobin, g/dL, Median [IQR]	11.8 [10.22-12.9]	11.82 [10.44-12.91]	11.72 [10.57-12.9]	11.68 [9.5-12.7]	0.294	
LDH, U/L, Median [IQR]	500.0 [432.0-667.25]	472.5 [388.0-556.29]	498.5 [439.19-649.58]	560.75 [460.25-788.0]	0.001**,§	Mild vs Intermediate, Severe
Lactic acid level, mmol/L, Mean [CI]	1.6 [1.52,1.68]	1.61 [1.48,1.75]	1.5 [1.38,1.63]	1.67 [1.53,1.82]	0.197	
Lymphocyte percentage, %, Median [IQR]	7.58 [4.53-12.1]	7.3 [4.65-11.9]	8.37 [5.28-12.8]	6.95 [3.91-11.88]	0.367	
Lymphocyte count, x10(9)/L, Median [IQR]	0.8 [0.5-1.18]	0.68 [0.49-1.09]	0.9 [0.55-1.22]	0.75 [0.49-1.17]	0.098	
Neutrophil percentage, %, Median [IQR]	86.0 [80.0-89.86]	85.8 [78.6-89.77]	85.25 [79.68-89.26]	86.97 [80.4-90.69]	0.301	
Neutrophil count, x10(9)/L, Median [IQR]	9.19 [6.79-12.43]	7.91 [6.21-10.65]	9.11 [7.15-12.49]	9.78 [7.12-13.84]	0.017**,§	Mild vs Intermediate, Severe
Platelet, x10(9)/L, Median [IQR]	249.5 [182.25-334.5]	251.5 [193.5-326.75]	266.0 [211.75-363.75]	230.0 [169.12-300.75]	0.003**,§	Intermediate vs Severe
Potassium, mmol/L, Median [IQR]	4.27 [3.9-4.65]	4.18 [3.8-4.5]	4.26 [3.89-4.54]	4.4 [3.9-4.93]	0.009**,§	Severe vs Mild, Intermediate

Procalcitonin, ng/mL, Median [IQR]	0.74 [0.31-2.25]	0.43 [0.17-0.9]	0.52 [0.27-1.05]	1.92 [0.63-7.01]	<0.001**,§	Severe vs Mild, Intermediate
Sodium, mmol/L, Median [IQR]	140.0 [136.27-142.5]	140.58 [136.88-142.62]	139.33 [137.0-142.0]	139.5 [136.0-143.0]	0.402	
Troponin, ng/mL, Mean [CI]	1.45 [0.52,2.37]	0.38 [0.08,0.68]	0.46 [0.08,0.84]	3.0 [0.71,5.29]	0.001**,§	Mild vs Intermediate, Severe
Triglycerides, mg/dL, Median [IQR]	224.5 [156.12-296.75]	237.0 [147.5-291.0]	218.12 [161.5-296.75]	224.5 [162.5-300.25]	0.997	
White blood cell, x10(9)/L, Median [IQR]	11.18 [8.1-14.9]	10.4 [7.07-12.49]	11.07 [8.45-14.95]	12.12 [8.96-15.8]	0.009**	Mild vs Intermediate, Severe
GCS, Mean [CI]	4.04 [3.79,4.29]	5.2 [4.4,6.0]	4.14 [3.78,4.5]	3.25 [3.1,3.41]	<0.001**,§	Severe vs Mild, Intermediate
MAP, mmHg, Median [IQR]	82.26 [76.62-88.13]	83.9 [78.21-91.99]	82.6 [77.19-87.06]	80.89 [75.5-87.74]	0.028§	Mild vs Severe
Temperature, degrees C, Median [IQR]	37.04 [36.58-37.66]	37.06 [36.6-37.76]	37.08 [36.7-37.68]	36.97 [36.53-37.56]	0.314	
Urine output, mL, Median [IQR]	1115.0 [741.25-1615.0]	1210.0 [942.5-1761.25]	1332.5 [921.25-1703.75]	840.5 [391.75-1264.75]	<0.001**,§	Severe vs Mild, Intermediate
FiO2, %, Median [IQR]	64.65 [53.59-74.47]	63.74 [51.09-74.34]	66.12 [56.01-73.11]	64.74 [54.41-75.17]	0.693	
PaO2, mmHg, Median [IQR]	89.0 [76.67-108.83]	89.08 [77.0-112.31]	86.28 [76.47-105.12]	91.42 [75.99-108.83]	0.733	
P/F ratio, Median [IQR]	144.02 [114.24-183.91]	152.0 [122.08-200.9]	137.81 [114.55-178.16]	142.89 [111.56-183.35]	0.406	
SpO2, Median [IQR]	96.1 [94.44-97.44]	96.63 [94.53-97.69]	95.78 [94.32-97.28]	95.75 [94.49-97.28]	0.280	
Driving pressure, Median [IQR]	13.17 [11.0-15.86]	13.08 [9.73-15.04]	13.9 [11.9-16.54]	13.17 [11.54-15.19]	0.038	Mild vs Intermediate
Minute ventilation, L, Median [IQR]	9.98 [8.6-11.3]	9.95 [8.69-10.46]	9.77 [8.52-11.12]	10.09 [8.75-11.83]	0.178	
PCO2, mmHg, Median [IQR]	45.92 [40.75-52.38]	44.9 [39.88-48.31]	46.14 [41.0-53.12]	46.86 [40.6-53.0]	0.194	
PEEP, Median [IQR]	10.83 [9.45-12.73]	10.0 [8.6-12.12]	11.0 [10.0-12.88]	11.1 [9.33-12.73]	0.213	
PH, Mean [CI]	7.37 [7.36,7.37]	7.37 [7.37,7.38]	7.37 [7.37,7.38]	7.36 [7.35,7.37]	0.029§	Severe vs Mild, Intermediate
PIP, Median [IQR]	30.0 [26.0-33.33]	29.33 [23.92-32.5]	29.83 [26.0-34.21]	30.37 [26.89-33.3]	0.017§	Mild vs Severe
Plateau pressure, Median [IQR]	25.0 [22.0-28.0]	24.0 [20.38-27.0]	25.5 [23.0-28.0]	25.0 [22.0-28.0]	0.066	
Static compliance, Median [IQR]	29.67 [23.22-37.02]	31.47 [24.59-43.55]	28.57 [23.07-35.0]	29.71 [21.7-37.31]	0.083	
Tidal PBW ratio, Median [IQR]	6.68 [6.34-7.52]	6.68 [6.48-7.52]	6.76 [6.35-7.7]	6.68 [6.29-7.3]	0.439	
Tidal volume, mL, Median [IQR]	433.67 [397.66-473.75]	437.92 [405.62-465.0]	425.36 [380.0-468.12]	445.5 [390.62-477.25]	0.608	
Ventilator ratio, Median [IQR]	2.04 [1.64-2.36]	1.98 [1.58-2.1]	1.93 [1.58-2.35]	2.1 [1.69-2.45]	0.144	

† p-value calculated by analysis of variance (ANOVA)/Kruskal–Wallis test
** False discovery rate corrected p-value < 0.05
§ Age adjusted (analysis of covariance [ANCOVA]) p-value < 0.05
Abbreviations: ALT=Alanine aminotransferase, AST=Aspartate aminotransferase, CI=confidence interval, CK=Creatine kinase, CRP=C-reactive protein, ESR=Erythrocyte sedimentation rate, FiO2=fraction of inspired oxygen, GCS=Glasgow Coma Scale, IQR=Interquartile range, LDH=Lactate dehydrogenase, MAP=Mean arterial pressure,

PaO2=partial pressure of oxygen, PBW=predicted body weight, PCO2=arterial partial pressure of carbon dioxide, PEEP=Positive end-expiratory pressure, PIP=Peak inspiratory pressure, P/F ratio=PaO2/FiO2 ratio, SpO2=oxygen saturation.

Table S6. Clinical variables (laboratory test results, vital signs, respiratory variables) of the baseline strata in NYP-LMH cohort. Data were examined at day 1 post-intubation.

Variable	All	Mild stratum	Intermediate stratum	Severe stratum	p-value†	Post-hoc
ALT, IU/L, Median [IQR]	38.0 [24.0-61.25]	64.5 [49.0-77.5]	33.0 [21.75-49.17]	40.0 [24.0-61.25]	0.045	Mild vs Intermediate
Albumin, g/dL, Median [IQR]	3.1 [2.72-3.3]	3.1 [3.06-3.24]	3.1 [2.7-3.47]	3.0 [2.7-3.2]	0.211	
AST, IU/L, Median [IQR]	54.0 [39.25-79.0]	58.0 [47.5-68.94]	53.0 [36.5-73.0]	51.33 [37.0-93.67]	0.782	
Bilirubin, mg/dL, Median [IQR]	0.7 [0.5-0.9]	0.7 [0.55-0.88]	0.6 [0.5-0.8]	0.65 [0.45-1.1]	0.873	
Bicarbonate, mmol/L, Median [IQR]	20.42 [18.5-23.0]	20.5 [20.0-21.62]	22.0 [19.75-24.5]	19.8 [16.25-22.0]	0.016	Intermediate vs Severe
CK, U/L, Median [IQR]	210.0 [101.65-481.81]	287.5 [140.5-1026.75]	240.0 [101.1-426.83]	184.0 [110.17-466.0]	0.690	
Creatinine, mg/dL, Median [IQR]	1.4 [0.8-2.23]	1.0 [0.7-1.25]	0.9 [0.7-1.25]	2.3 [1.6-2.85]	<0.001**,§	Severe vs Mild, Intermediate
CRP, mg/dL, Median [IQR]	17.87 [15.12-24.52]	16.27 [15.5-17.55]	18.19 [14.68-25.12]	18.61 [15.05-24.14]	0.661	
D-dimer, ng/mL, Median [IQR]	1508.0 [979.38-2955.53]	1329.0 [679.38-1508.0]	1470.0 [710.0-2457.54]	1747.0 [1207.25-3857.5]	0.053	
ESR, mm/hr, Median [IQR]	88.0 [69.88-98.92]	88.0 [79.75-88.0]	88.0 [73.0-97.5]	88.0 [64.0-109.0]	0.954	
Ferritin, ng/mL, Median [IQR]	1242.57 [889.56-1901.14]	1330.89 [1099.24-2126.9]	1426.04 [995.66-1935.52]	1185.5 [702.08-1595.1]	0.430	
Globulin, g/dL, Median [IQR]	3.0 [3.0-3.5]	3.0 [2.5-3.0]	3.0 [3.0-3.5]	3.0 [3.0-3.5]	0.331	
Glucose, mg/dL, Median [IQR]	150.5 [114.88-196.62]	150.0 [123.31-188.75]	121.0 [100.5-171.25]	172.0 [131.33-228.95]	0.005§	Intermediate vs Severe
Hemoglobin, g/dL, Median [IQR]	12.05 [11.1-13.25]	12.62 [12.03-13.03]	11.9 [11.18-12.95]	12.27 [10.8-13.62]	0.684	
LDH, U/L, Median [IQR]	518.39 [437.12-741.75]	546.71 [485.88-678.25]	504.0 [430.42-673.5]	561.5 [437.17-784.75]	0.719	
Lactic acid level, mmol/L, Median [IQR]	2.2 [1.88,2.52]	2.95 [1.29,4.62]	2.01 [1.55,2.47]	2.17 [1.75,2.6]	0.312	
Lymphocyte percentage, %, Median [IQR]	8.15 [4.64-13.29]	7.55 [5.12-11.67]	9.55 [7.33-15.0]	5.9 [3.92-11.47]	0.008	Intermediate vs Severe
Lymphocyte count, x10(9)/L, Median [IQR]	0.74 [0.53-1.06]	0.7 [0.6-0.88]	0.81 [0.63-1.2]	0.7 [0.46-1.0]	0.225	
Neutrophil percentage, %, Median [IQR]	86.88 [80.45-91.14]	87.65 [82.46-90.7]	85.4 [79.6-87.38]	90.05 [83.0-92.05]	0.007	Intermediate vs Severe
Neutrophil count, x10(9)/L, Median [IQR]	8.88 [6.27-11.34]	8.76 [6.54-12.22]	7.75 [5.59-9.61]	10.37 [6.73-12.81]	0.013§	Intermediate vs Severe
Platelet, x10(9)/L, Median [IQR]	236.0 [187.75-312.25]	202.5 [188.25-292.5]	264.0 [188.0-317.5]	221.0 [177.5-303.0]	0.463	
Potassium, mmol/L, Median [IQR]	4.2 [3.8-4.67]	4.38 [3.95-4.66]	4.0 [3.7-4.35]	4.23 [3.92-5.05]	0.016§	Intermediate vs Severe

Procalcitonin, ng/mL, Median [IQR]	0.8 [0.3-1.82]	0.36 [0.15-0.57]	0.49 [0.17-2.01]	1.11 [0.46-1.84]	0.038	Mild vs Severe
Sodium, mmol/L, Median [IQR]	138.25 [135.0-141.54]	139.88 [136.25-142.75]	139.0 [134.0-141.0]	138.0 [135.5-140.75]	0.693	
Troponin, ng/mL, Mean [CI]	0.33 [0.13,0.54]	0.04 [0.0,0.08]	0.31 [0.02,0.61]	0.43 [0.06,0.8]	0.071	
Triglycerides, mg/dL, Median [IQR]	228.0 [157.25-311.25]	229.0 [228.0-310.14]	228.0 [160.0-301.0]	228.0 [152.0-309.0]	0.566	
White blood cell, x10(9)/L, Median [IQR]	10.8 [7.79-13.56]	8.95 [6.9-13.43]	10.4 [7.45-11.9]	12.4 [8.6-14.45]	0.081	
GCS, Mean [CI]	5.36 [4.81,5.91]	7.8 [4.83,10.77]	5.51 [4.87,6.16]	4.59 [3.9,5.28]	0.010[§]	Severe vs Mild, Intermediate
MAP, mmHg, Median [IQR]	82.91 [77.78-86.68]	83.96 [78.58-91.71]	82.52 [76.82-85.95]	84.8 [79.18-88.49]	0.238	
Temperature, degrees C, Median [IQR]	37.19 [36.7-37.64]	37.13 [36.46-37.62]	37.42 [36.95-37.73]	37.03 [36.63-37.43]	0.121	
Urine output, mL, Median [IQR]	947.5 [601.25-1400.0]	1227.5 [1119.38-1612.5]	1120.0 [882.5-1555.0]	620.0 [310.0-952.5]	<0.001[**,§]	Severe vs Mild, Intermediate
FiO2, %, Median [IQR]	77.88 [66.67-88.87]	68.74 [59.2-87.44]	75.0 [62.0-89.38]	80.0 [71.88-89.75]	0.157	
PaO2, mmHg, Median [IQR]	111.04 [89.5-134.83]	105.69 [96.09-129.74]	98.62 [86.5-122.92]	118.0 [95.58-138.09]	0.238	
P/F ratio, Median [IQR]	143.07 [112.48-190.51]	151.72 [144.65-176.9]	136.5 [107.88-195.83]	141.9 [118.78-188.27]	0.412	
SpO2, Median [IQR]	96.53 [95.14-97.85]	95.75 [93.31-98.14]	96.59 [94.52-97.6]	96.82 [95.31-97.89]	0.357	
Driving pressure, Median [IQR]	12.37 [9.83-14.76]	12.58 [10.87-15.75]	13.0 [8.67-14.88]	12.0 [9.64-14.25]	0.613	
Minute ventilation, L, Median [IQR]	9.17 [8.26-10.87]	9.52 [8.73-10.72]	9.01 [8.3-10.99]	9.18 [7.99-10.73]	0.966	
PCO2, mmHg, Median [IQR]	42.21 [37.95-46.88]	38.82 [33.89-44.24]	41.48 [38.0-46.16]	43.9 [38.44-48.05]	0.220	
PEEP, Median [IQR]	11.65 [9.62-13.38]	10.64 [8.5-12.04]	12.0 [9.33-13.25]	11.86 [9.86-13.48]	0.607	
PH, Mean [CI]	7.34 [7.33,7.36]	7.37 [7.33,7.41]	7.38 [7.36,7.4]	7.31 [7.29,7.33]	<0.001[**,§]	Severe vs Mild, Intermediate
PIP, Median [IQR]	27.9 [24.67-31.0]	26.81 [25.0-28.5]	28.0 [25.13-31.45]	28.0 [24.73-31.0]	0.377	
Plateau pressure, Median [IQR]	24.0 [19.75-27.0]	24.83 [23.38-25.75]	24.0 [21.5-28.0]	24.0 [18.33-25.75]	0.555	
Static compliance, Median [IQR]	28.0 [23.24-35.22]	28.88 [26.49-36.14]	26.43 [22.58-32.14]	29.68 [23.88-36.52]	0.312	
Tidal PBW ratio, Median [IQR]	6.51 [6.04-7.08]	6.6 [6.1-7.03]	6.52 [6.29-7.48]	6.41 [5.94-6.6]	0.126	
Tidal volume, mL, Median [IQR]	400.0 [370.0-440.62]	400.0 [385.0-417.08]	400.0 [370.0-450.0]	400.0 [350.0-425.0]	0.758	
Ventilator ratio, Median [IQR]	1.83 [1.56-2.01]	1.71 [1.3-1.94]	1.87 [1.56-2.0]	1.83 [1.62-2.02]	0.451	

† p-value calculated by analysis of variance (ANOVA)/Kruskal–Wallis test
** False discovery rate corrected p-value < 0.05
§ Age adjusted (analysis of covariance [ANCOVA]) p-value < 0.05
Abbreviations: ALT=Alanine aminotransferase, AST=Aspartate aminotransferase, CI=confidence interval, CK=Creatine kinase, CRP=C-reactive protein, ESR=Erythrocyte sedimentation rate, FiO2=fraction of inspired oxygen, GCS=Glasgow Coma Scale, IQR=Interquartile range, LDH=Lactate dehydrogenase, MAP=Mean arterial pressure,

PaO_2=partial pressure of oxygen, PBW=predicted body weight, PCO_2=arterial partial pressure of carbon dioxide, PEEP=Positive end-expiratory pressure, PIP=Peak inspiratory pressure, P/F ratio=PaO_2/FiO_2 ratio, SpO_2=oxygen saturation.

Table S7. Clinical variables (laboratory test results, vital signs, respiratory variables, and ventilator parameters) of the trajectory subphenotypes in NYP-WCMC cohort. Data were examined at day 1 and day 3 post-intubation.

		Mild stratum			Intermediate stratum			Severe stratum		
Variable	Time	Worsening	Recovering	p-value†	Worsening	Recovering	p-value†	Worsening	Recovering	p-value†
ALT, IU/L, Median [IQR]	day 1	47.0 [25.0-58.5]	33.67 [21.0-62.0]	0.167	45.5 [29.0-87.0]	40.0 [26.5-66.5]	0.107	47.5 [29.25-70.5]	43.5 [22.0-78.12]	0.203
	day 3	49.0 [27.0-61.0]	41.0 [28.5-56.0]	0.250	42.5 [24.0-66.0]	41.0 [24.5-62.0]	0.457	47.0 [24.25-92.0]	41.0 [22.0-66.25]	0.090
Albumin, g/dL, Median [IQR]	day 1	2.2 [1.9-2.45]	2.2 [2.0-2.55]	0.479	2.25 [1.8-2.5]	2.2 [2.0-2.4]	0.863	2.1 [1.75-2.45]	2.0 [1.7-2.41]	0.396
	day 3	1.9 [1.5-2.1]	2.0 [1.73-2.35]	0.079	1.8 [1.5-1.9]	1.9 [1.66-2.09]	0.025	1.62 [1.42-1.89]	1.75 [1.6-2.0]	0.036
AST, IU/L, Median [IQR]	day 1	44.0 [34.5-58.5]	39.5 [33.0-61.5]	0.324	58.0 [39.0-98.0]	43.0 [33.75-58.0]	0.014§	68.5 [43.62-115.62]	56.5 [34.0-94.75]	0.041
	day 3	53.67 [33.33-72.0]	41.0 [30.0-65.0]	0.136	45.0 [30.0-86.0]	41.75 [25.05-71.0]	0.153	63.0 [38.75-128.5]	44.5 [33.0-73.5]	0.012**
Bilirubin, mg/dL, Median [IQR]	day 1	0.6 [0.5-1.1]	0.6 [0.4-0.8]	0.127	0.8 [0.5-1.0]	0.6 [0.45-0.9]	0.122	0.8 [0.5-1.25]	0.72 [0.5-1.2]	0.395
	day 3	0.6 [0.47-1.0]	0.6 [0.4-0.8]	0.280	0.6 [0.4-1.1]	0.5 [0.3-0.8]	0.061§	0.85 [0.5-1.48]	0.7 [0.47-1.1]	0.052
Bicarbonate, mmol/L, Median [IQR]	day 1	26.0 [25.0-28.5]	27.0 [25.0-29.25]	0.178	26.0 [23.0-28.0]	26.33 [23.42-29.0]	0.541	24.0 [21.0-25.92]	24.0 [22.0-26.62]	0.404
	day 3	28.0 [26.0-31.33]	29.0 [26.38-31.0]	0.545	28.0 [23.0-29.5]	29.0 [27.0-32.0]	0.001**,§	24.0 [21.5-26.25]	25.25 [22.25-29.0]	0.038§
CK, U/L, Median [IQR]	day 1	187.0 [72.0-346.0]	160.0 [59.5-215.5]	0.054	178.0 [80.0-330.4]	145.0 [78.62-263.0]	0.190	198.0 [115.38-439.25]	170.5 [73.0-368.0]	0.058
	day 3	187.0 [66.0-413.0]	125.67 [79.0-204.55]	0.054§	163.0 [74.5-366.8]	170.0 [71.83-362.0]	0.385§	276.5 [140.04-645.31]	156.0 [79.0-423.0]	0.016**
Creatinine, mg/dL, Median [IQR]	day 1	0.89 [0.73-1.09]	0.77 [0.64-0.9]	0.004	1.12 [0.83-1.36]	0.93 [0.74-1.18]	0.025	2.17 [1.45-3.86]	1.75 [1.22-2.99]	0.037
	day 3	0.9 [0.71-1.49]	0.72 [0.59-0.9]	0.001**,§	1.34 [0.84-2.25]	0.95 [0.74-1.14]	0.001**,§	3.59 [1.76-4.48]	1.74 [1.09-3.14]	<0.001**,§
CRP, mg/dL, Median [IQR]	day 1	22.1 [12.76-29.0]	16.96 [8.75-23.21]	0.038§	20.4 [13.4-27.9]	21.0 [15.2-26.98]	0.543	19.32 [14.35-27.7]	21.47 [12.6-28.44]	0.478
	day 3	22.2 [10.68-27.5]	16.8 [8.75-20.59]	0.042§	23.7 [18.35-30.73]	20.22 [13.38-25.25]	0.029§	20.75 [14.55-29.15]	18.95 [11.67-26.62]	0.144
D-dimer, ng/mL, Median [IQR]	day 1	908.0 [527.86-2384.0]	1331.75 [648.25-2006.5]	0.250	1331.75 [677.0-3853.0]	1331.75 [701.25-2198.08]	0.232	1866.08 [1041.35-3862.25]	1964.38 [946.75-4933.88]	0.400
	day 3	1872.33 [591.0-2994.2]	971.0 [749.5-2103.0]	0.184	1482.0 [847.0-3015.0]	1331.75 [746.0-2167.0]	0.211	2135.0 [1140.0-3351.5]	1888.75 [841.7-3252.25]	0.099
ESR, mm/hr, Median [IQR]	day 1	83.0 [75.0-105.0]	83.0 [65.5-97.06]	0.321	83.0 [68.4-83.0]	83.0 [66.64-90.12]	0.964	83.0 [60.78-99.9]	83.0 [74.5-104.8]	0.113

	day 3	83.0 [78.0-100.0]	83.0 [73.0-98.5]	0.413	83.0 [65.0-88.5]	83.0 [68.0-92.0]	0.488	81.94 [58.54-93.38]	83.0 [72.5-99.66]	0.046
Ferritin, ng/mL, Median [IQR]	day 1	1143.3 [596.2-1525.3]	1073.35 [660.0-1653.23]	0.461	1166.3 [644.1-1638.8]	1154.5 [622.88-1431.0]	0.227	1444.21 [1166.3-2155.03]	1166.3 [813.15-1598.56]	0.018
	day 3	1166.3 [751.15-1565.6]	1066.55 [712.0-1748.48]	0.360	1166.3 [635.34-1702.3]	1166.3 [659.01-1497.55]	0.315	1459.11 [1166.3-2394.41]	1166.3 [848.4-1660.47]	0.016**
Globulin, g/dL, Median [IQR]	day 1	3.45 [3.2-3.7]	3.3 [3.1-3.52]	0.026	3.5 [3.0-3.8]	3.4 [3.2-3.8]	0.346	3.38 [2.9-3.88]	3.45 [3.04-3.86]	0.447
	day 3	3.6 [3.4-3.9]	3.4 [2.95-3.7]	0.033	3.3 [3.0-3.65]	3.5 [3.2-3.8]	0.080	3.3 [2.92-3.6]	3.5 [3.0-3.81]	0.271
Glucose, mg/dL, Median [IQR]	day 1	122.0 [104.0-147.0]	116.0 [104.33-144.0]	0.284	148.0 [110.0-175.5]	144.0 [121.67-191.5]	0.392	159.17 [131.96-233.5]	152.17 [122.25-197.5]	0.103
	day 3	144.75 [119.0-182.67]	127.0 [112.25-146.5]	0.023	161.0 [118.0-192.5]	142.0 [122.5-181.5]	0.333	160.0 [129.88-215.38]	156.67 [130.38-198.12]	0.375
Hemoglobin, g/dL, Median [IQR]	day 1	12.35 [10.45-13.05]	11.8 [10.35-12.85]	0.401	11.8 [10.3-12.85]	11.65 [10.7-12.9]	0.876	11.6 [9.27-12.4]	11.83 [9.79-12.93]	0.073
	day 3	11.1 [10.2-12.7]	11.0 [10.12-12.05]	0.588	10.8 [9.7-11.9]	10.8 [9.85-12.0]	0.597	9.65 [8.1-11.47]	10.45 [9.0-11.51]	0.046
LDH, U/L, Median [IQR]	day 1	483.4 [378.0-592.0]	465.0 [399.5-526.75]	0.175	536.0 [455.0-785.0]	490.5 [430.5-607.0]	0.046§	589.0 [467.25-882.88]	519.75 [420.25-728.41]	0.028
	day 3	474.0 [389.0-569.67]	437.0 [391.17-494.5]	0.070	474.33 [402.0-620.5]	442.0 [374.5-521.5]	0.010**,§	547.5 [444.75-760.92]	482.17 [384.75-619.82]	0.016**
Lactic acid level, mmol/L, Mean [IC]	day 1	1.68 [1.46,1.9]	1.56 [1.4,1.72]	0.442	1.45 [1.25,1.64]	1.54 [1.38,1.69]	0.206	1.76 [1.53,1.99]	1.61 [1.43,1.79]	0.068
	day 3	1.63 [1.42,1.85]	1.53 [1.38,1.68]	0.427	1.38 [1.24,1.51]	1.54 [1.38,1.69]	0.203	1.74 [1.51,1.98]	1.56 [1.39,1.74]	0.046
Lymphocyte percentage, %, Median [IQR]	day 1	6.55 [4.4-10.2]	7.6 [5.4-15.0]	0.101	6.8 [4.7-12.47]	8.9 [6.32-12.85]	0.091	7.48 [3.7-11.73]	6.72 [4.14-11.93]	0.364
	day 3	7.37 [4.1-9.6]	8.8 [5.22-14.05]	0.084	6.2 [5.1-8.9]	9.0 [6.5-12.3]	0.008**	5.9 [3.75-8.05]	7.35 [4.39-11.1]	0.035
Lymphocyte count, x10(9)/L, Median [IQR]	day 1	0.74 [0.48-1.12]	0.67 [0.49-1.08]	0.498	0.8 [0.48-1.21]	0.95 [0.58-1.24]	0.190	0.73 [0.39-0.97]	0.81 [0.55-1.27]	0.041
	day 3	0.84 [0.47-1.12]	0.73 [0.57-1.06]	0.313	0.88 [0.6-0.97]	0.87 [0.58-1.24]	0.300	0.58 [0.37-0.84]	0.73 [0.48-1.1]	0.023
Neutrophil percentage, %, Median [IQR]	day 1	86.1 [82.0-89.97]	84.7 [76.65-89.4]	0.147	86.35 [80.8-89.3]	84.67 [79.25-89.15]	0.197	86.92 [80.41-91.15]	87.3 [80.72-90.4]	0.351
	day 3	83.9 [78.85-90.2]	83.0 [75.8-89.33]	0.169	86.4 [83.05-89.0]	83.5 [78.05-86.9]	0.014	87.0 [83.88-91.62]	86.1 [80.29-89.11]	0.067
Neutrophil count, x10(9)/L, Median [IQR]	day 1	9.59 [7.22-11.15]	7.04 [4.87-10.28]	0.013	10.64 [7.12-13.44]	8.81 [7.16-12.25]	0.178	9.4 [5.95-12.26]	10.02 [7.64-14.9]	0.077
	day 3	9.34 [7.57-13.48]	7.71 [5.19-10.18]	0.005**,§	10.26 [8.01-12.38]	7.63 [6.18-10.42]	0.001**,§	9.23 [5.59-12.23]	8.88 [6.85-11.61]	0.499

Platelet, x10(9)/L, Median [IQR]	day 1	280.0 [201.0-367.0]	218.0 [175.5-310.5]	0.039[§]	268.0 [223.0-348.0]	259.0 [206.5-368.5]	0.475	212.5 [169.62-276.75]	253.5 [169.25-335.25]	0.027[§]
	day 3	286.0 [253.0-379.0]	268.0 [193.5-335.0]	0.144	310.0 [233.0-363.0]	282.0 [210.75-384.0]	0.401	188.5 [145.25-257.0]	242.0 [165.75-295.0]	0.006[**,§]
Potassium, mmol/L, Median [IQR]	day 1	4.1 [3.8-4.4]	4.25 [3.85-4.5]	0.202	4.33 [3.9-4.55]	4.2 [3.88-4.5]	0.280	4.38 [4.06-4.94]	4.4 [3.84-4.91]	0.241
	day 3	4.4 [4.05-4.97]	4.2 [3.8-4.57]	0.149	4.35 [4.0-4.6]	4.2 [3.88-4.5]	0.126	4.33 [4.0-5.01]	4.2 [3.9-4.6]	0.037
Procalcitonin, ng/mL, Median [IQR]	day 1	0.67 [0.32-1.33]	0.33 [0.16-0.77]	0.017	0.51 [0.27-1.1]	0.57 [0.27-1.03]	0.385	2.72 [0.9-7.76]	1.34 [0.48-4.99]	0.034
	day 3	0.75 [0.32-1.76]	0.28 [0.17-0.73]	0.002[**,§]	0.88 [0.52-2.06]	0.68 [0.29-1.84]	0.073	3.21 [1.28-13.22]	1.47 [0.6-5.67]	0.005[**]
Sodium, mmol/L, Median [IQR]	day 1	141.5 [138.5-143.0]	140.0 [136.42-142.0]	0.061	139.67 [137.0-142.0]	139.0 [137.0-142.25]	0.385	138.67 [135.5-142.25]	140.0 [136.46-143.0]	0.288
	day 3	142.5 [140.5-145.0]	140.0 [138.0-143.0]	0.009[§]	141.0 [137.5-145.0]	143.0 [140.5-145.0]	0.037[§]	140.0 [136.17-144.38]	142.0 [138.0-145.62]	0.064
Troponin, ng/mL, Mean [CI]	day 1	0.53 [-0.05,1.12]	0.24 [0.05,0.43]	0.123	0.39 [0.06,0.72]	0.5 [-0.06,1.06]	0.499	4.4 [-0.5,9.31]	1.95 [0.24,3.65]	0.032
	day 3	0.39 [-0.13,0.91]	0.18 [0.03,0.34]	0.120	0.21 [0.1,0.33]	0.41 [-0.1,0.91]	0.442	4.8 [-0.42,10.02]	1.23 [0.27,2.2]	0.030
Triglycerides, mg/dL, Median [IQR]	day 1	237.0 [141.0-365.5]	233.0 [155.5-237.0]	0.139	224.0 [192.0-313.0]	203.0 [151.5-291.0]	0.094	237.0 [177.0-311.62]	205.75 [145.0-272.5]	0.045
	day 3	237.0 [181.5-538.0]	233.0 [185.25-237.0]	0.080	239.0 [210.0-335.0]	251.0 [185.0-329.0]	0.231[§]	269.17 [208.55-436.75]	237.0 [156.5-311.25]	0.016[**]
WBC, x10(9)/L, Median [IQR]	day 1	11.2 [8.45-13.1]	8.73 [6.1-11.45]	0.013	11.9 [8.8-16.1]	10.85 [8.37-14.6]	0.219	12.0 [7.14-14.67]	12.4 [9.91-16.6]	0.142
	day 3	12.3 [9.1-15.3]	9.6 [6.65-11.15]	0.001[**,§]	12.0 [8.9-14.45]	9.4 [7.35-11.9]	0.005[**,§]	12.0 [7.32-16.28]	10.4 [8.05-13.54]	0.164
GCS, Median [IQR]	day 1	5.16 [3.98,6.34]	5.23 [4.1,6.36]	0.374	4.2 [3.62,4.77]	4.11 [3.63,4.58]	0.334	3.26 [3.04,3.48]	3.25 [3.04,3.46]	0.246
	day 3	4.27 [3.41,5.13]	7.26 [5.87,8.66]	<0.001[**,§]	3.44 [3.08,3.8]	4.76 [4.14,5.38]	0.003[**,§]	3.46 [3.16,3.77]	4.62 [3.97,5.28]	0.012
MAP, mmHg, Median [IQR]	day 1	85.71 [80.0-94.16]	82.0 [77.46-89.44]	0.174	82.25 [76.67-87.5]	83.0 [77.67-86.9]	0.386	82.17 [76.35-87.86]	80.06 [74.73-86.35]	0.161
	day 3	80.76 [78.5-88.0]	85.74 [78.46-93.77]	0.163	81.3 [72.5-87.5]	83.75 [77.0-90.36]	0.059	82.35 [74.81-87.03]	81.88 [78.77-86.15]	0.277
Temperature, degrees C, Median [IQR]	day 1	37.2 [36.48-37.69]	37.02 [36.66-37.84]	0.436	36.94 [36.54-37.6]	37.14 [36.71-37.7]	0.162	36.94 [36.35-37.45]	37.02 [36.63-37.61]	0.116
	day 3	37.28 [36.47-37.8]	36.92 [36.61-37.36]	0.267	36.98 [36.55-37.47]	37.2 [36.83-37.66]	0.110	36.74 [36.09-37.08]	36.83 [36.48-37.39]	0.047[§]
Urine output, mL, Median [IQR]	day 1	1050.0 [830.0-1695.0]	1365.0 [1055.0-1800.0]	0.093	1065.0 [840.0-1715.0]	1350.0 [1030.0-1650.0]	0.085	815.5 [385.0-1285.0]	885.0 [395.25-1264.25]	0.429
	day 3	1930.0 [1125.0-2263.0]	1665.0 [1130.0-2052.0]	0.201	1290.0 [900.0-2150.0]	1860.0 [1315.0-2627.5]	0.005[**]	1040.0 [240.0-1700.0]	1197.5 [721.0-1706.25]	0.097
FiO2, %, Median [IQR]	day 1	65.62 [50.0-74.24]	63.44 [51.92-73.99]	0.667	69.62 [62.58-75.0]	62.68 [52.47-71.35]	0.036	67.08 [57.93-76.64]	63.14 [53.79-74.41]	0.338
	day 3	50.94 [40.0-60.0]	45.0 [40.0-54.36]	0.158	50.0 [43.7-60.0]	44.23 [39.17-50.0]	0.001[**,§]	50.34 [45.77-64.04]	45.82 [40.0-55.05]	0.004[**,§]

PaO2, mmHg, Median [IQR]	day 1	86.5 [75.71-103.29]	90.75 [77.0-117.02]	0.126	92.0 [76.5-114.44]	85.5 [76.58-100.79]	0.122	89.0 [73.97-103.58]	95.07 [80.38-114.08]	0.023
	day 3	80.6 [70.0-86.75]	81.0 [71.33-92.65]	0.372	79.0 [68.0-96.82]	77.0 [71.12-85.38]	0.350	74.53 [65.55-83.36]	80.78 [68.75-94.3]	0.055
P/F ratio, Median [IQR]	day 1	147.41 [110.72-192.41]	154.0 [124.68-211.47]	0.253	134.23 [113.64-183.46]	143.68 [116.18-173.9]	0.452	129.13 [100.39-170.93]	149.49 [123.13-214.85]	0.016
	day 3	152.78 [128.33-209.17]	189.57 [148.37-213.75]	0.142	163.09 [125.68-190.59]	178.39 [147.29-217.5]	0.009[**,§]	145.5 [118.15-171.6]	176.55 [140.53-209.65]	0.001[**,§]
SpO2, Median [IQR]	day 1	96.64 [94.44-97.55]	96.61 [95.18-98.23]	0.140	95.91 [95.07-97.48]	95.47 [94.26-97.19]	0.060	95.43 [94.01-96.59]	96.22 [94.53-97.63]	0.011
	day 3	95.4 [93.27-97.2]	95.95 [94.46-98.0]	0.086	95.83 [93.74-96.76]	95.24 [93.72-96.89]	0.978	95.18 [93.52-96.69]	95.9 [94.48-97.32]	0.109
Driving pressure, Median [IQR]	day 1	13.17 [10.0-16.0]	13.0 [9.33-13.45]	0.135	14.5 [11.0-17.0]	13.67 [11.96-16.0]	0.253	13.08 [11.0-15.97]	13.67 [11.73-15.0]	0.419
	day 3	12.24 [10.0-16.0]	13.0 [10.0-13.58]	0.399	13.5 [10.5-17.0]	13.17 [11.17-15.8]	0.682	14.0 [11.81-18.0]	13.17 [10.0-15.11]	0.014[**,§]
Minute ventilation, L, Median [IQR]	day 1	10.3 [8.9-11.63]	9.53 [8.45-10.12]	0.006[§]	10.17 [8.59-11.05]	9.48 [8.51-11.21]	0.255	10.21 [8.89-12.06]	10.04 [8.42-11.66]	0.201
	day 3	9.5 [8.45-11.95]	9.1 [7.93-10.07]	0.008[§]	10.15 [8.28-11.5]	9.2 [8.47-10.65]	0.510	11.3 [9.61-13.44]	10.21 [8.46-12.29]	0.006[**,§]
PCO2, mmHg, Median [IQR]	day 1	43.0 [38.33-47.25]	45.0 [41.68-49.0]	0.160	46.5 [43.0-52.0]	45.67 [40.8-53.58]	0.435	47.67 [42.71-53.3]	45.83 [40.18-52.57]	0.370
	day 3	47.0 [42.0-53.0]	46.33 [39.67-49.45]	0.050	47.67 [46.0-54.5]	49.0 [43.75-55.43]	0.980	45.75 [42.89-54.2]	44.75 [41.11-50.69]	0.156
PEEP, Median [IQR]	day 1	10.67 [9.6-13.0]	10.0 [8.33-11.8]	0.066	10.86 [8.29-12.5]	11.25 [10.0-12.92]	0.172	11.6 [10.0-12.73]	10.67 [8.65-12.7]	0.075
	day 3	11.0 [9.0-12.67]	10.0 [7.5-13.75]	0.148	12.0 [10.0-13.11]	11.0 [9.0-12.45]	0.104	12.0 [10.0-14.38]	11.8 [8.0-13.5]	0.012[**,§]
PH, Mean [CI]	day 1	7.38 [7.37,7.38]	7.37 [7.36,7.38]	0.359	7.37 [7.36,7.38]	7.38 [7.37,7.39]	0.150	7.36 [7.35,7.37]	7.36 [7.35,7.37]	0.475
	day 3	7.37 [7.36,7.38]	7.37 [7.36,7.38]	0.376	7.37 [7.36,7.38]	7.38 [7.37,7.39]	0.406	7.36 [7.35,7.37]	7.36 [7.35,7.37]	0.342
PIP, Median [IQR]	day 1	29.33 [24.5-33.0]	29.33 [23.83-30.83]	0.300	30.0 [25.33-34.5]	29.67 [26.62-34.07]	0.833	31.83 [28.44-35.48]	29.67 [26.25-32.0]	0.019
	day 3	28.5 [26.0-32.67]	27.17 [23.75-29.67]	0.055	29.5 [26.33-33.5]	28.0 [24.17-32.21]	0.061	32.75 [28.0-35.0]	28.9 [24.73-32.5]	0.001[**,§]
Plateau pressure, Median [IQR]	day 1	24.0 [20.0-28.0]	24.0 [20.75-25.0]	0.321	26.0 [22.5-29.0]	25.0 [23.42-28.0]	0.600	25.0 [22.25-29.46]	25.0 [21.75-28.0]	0.324
	day 3	23.75 [19.58-28.0]	24.0 [19.67-25.0]	0.243	25.33 [22.0-29.5]	25.0 [21.33-28.0]	0.296	26.83 [24.0-30.56]	25.0 [21.0-27.0]	0.001[**,§]
Static compliance, Median [IQR]	day 1	32.93 [24.7-43.19]	31.11 [25.02-41.93]	0.438	27.14 [20.09-34.66]	28.67 [24.66-35.08]	0.191	28.85 [20.92-37.76]	30.39 [21.86-35.81]	0.308
	day 3	35.59 [21.88-42.11]	31.59 [28.8-38.95]	0.874	27.1 [22.54-36.17]	28.72 [23.61-36.15]	0.292	25.8 [22.18-31.98]	29.69 [21.32-40.96]	0.071
Tidal PBW ratio, Median [IQR]	day 1	6.68 [6.22-7.32]	6.74 [6.68-7.9]	0.030	6.91 [6.35-7.7]	6.7 [6.36-7.59]	0.275	6.66 [6.17-7.13]	6.73 [6.41-7.46]	0.053
	day 3	6.61 [6.03-6.86]	6.68 [6.33-7.08]	0.132	6.67 [6.21-7.25]	6.68 [6.04-7.1]	0.424	6.63 [6.15-6.91]	6.47 [6.01-7.06]	0.434

Tidal volume, mL, Median [IQR]	day 1	450.0 [400.0-475.0]	425.0 [417.92-461.25]	0.444	435.0 [383.33-475.0]	425.0 [380.0-461.25]	0.308	436.67 [375.0-473.75]	450.0 [400.0-478.5]	0.384
	day 3	410.0 [380.0-450.0]	420.0 [372.5-455.0]	0.668	410.0 [366.67-456.67]	400.0 [375.83-450.0]	0.933	420.0 [362.5-477.5]	420.0 [380.0-452.5]	0.966
Ventilator ratio, Median [IQR]	day 1	1.89 [1.58-2.1]	2.06 [1.61-2.1]	0.261	2.2 [1.7-2.61]	1.87 [1.58-2.23]	0.070	2.22 [1.83-2.47]	2.0 [1.64-2.39]	0.059
	day 3	2.08 [1.71-2.47]	1.98 [1.58-2.1]	0.152	2.18 [1.71-2.46]	2.04 [1.67-2.47]	0.192	2.18 [1.91-2.77]	2.09 [1.58-2.48]	0.017[**,§]

[†] p-value calculated by student's t-test/Mann-Whitney test

** False discovery rate corrected p-value < 0.05

[§] Age adjusted (analysis of covariance [ANCOVA]) p-value < 0.05

Abbreviations: ALT=Alanine aminotransferase, AST=Aspartate aminotransferase, CI=confidence interval, CK=Creatine kinase, CRP=C-reactive protein, ESR=Erythrocyte sedimentation rate, FiO2=fraction of inspired oxygen, GCS=Glasgow Coma Scale, IQR=Interquartile range, LDH=Lactate dehydrogenase, MAP=Mean arterial pressure, PaO2=partial pressure of oxygen, PBW=predicted body weight, PCO2=arterial partial pressure of carbon dioxide, PEEP=Positive end-expiratory pressure, PIP=Peak inspiratory pressure, P/F ratio=PaO2/FiO2 ratio, SpO2=oxygen saturation.

Table S8. Clinical variables (laboratory test results, vital signs, respiratory variables) of the trajectory subphenotypes in NYP-LMH cohort. Data were examined at day 1 and day 3 post-intubation.

		Mild stratum			Intermediate stratum			Severe stratum		
Variable	Time	Worsening	Recovering	p-value†	Worsening	Recovering	p-value†	Worsening	Recovering	p-value†
ALT, IU/L, Median [IQR]	day 1	68.0 [51.0-75.0]	61.0 [42.5-80.0]	0.834	36.5 [30.0-49.75]	32.5 [19.0-45.0]	0.153	40.75 [27.0-61.12]	36.0 [22.62-61.0]	0.363
	day 3	46.0 [37.92-63.5]	45.0 [31.0-78.5]	0.685	32.5 [28.25-47.12]	25.0 [16.0-42.0]	0.119	35.5 [20.88-44.25]	35.0 [22.25-58.0]	0.446
Albumin, g/dL, Median [IQR]	day 1	3.1 [3.02-3.1]	3.25 [3.23-3.35]	0.055	3.1 [2.7-3.43]	3.0 [2.9-3.55]	0.854	3.05 [2.72-3.16]	2.9 [2.62-3.23]	0.373
	day 3	2.65 [2.44-3.05]	2.3 [2.15-2.5]	0.250	2.55 [2.4-2.8]	2.55 [2.3-2.9]	0.692	2.4 [1.9-2.81]	2.3 [1.95-2.6]	0.631
AST, IU/L, Median [IQR]	day 1	58.0 [48.0-58.5]	72.25 [59.12-81.12]	0.246	59.75 [49.25-76.62]	37.0 [30.0-65.0]	0.023	76.5 [47.0-101.0]	49.0 [33.5-80.0]	0.066
	day 3	58.0 [28.5-92.08]	37.0 [30.5-86.0]	0.912	52.5 [39.88-63.5]	47.0 [24.5-61.0]	0.116	64.5 [31.21-87.0]	43.0 [24.62-69.0]	0.081
Bilirubin, mg/dL, Median [IQR]	day 1	0.7 [0.6-0.95]	0.7 [0.55-0.75]	0.460	0.6 [0.53-0.8]	0.7 [0.5-0.8]	0.366	0.78 [0.57-1.3]	0.6 [0.4-0.95]	0.136
	day 3	0.6 [0.55-1.8]	0.5 [0.5-0.65]	0.244	0.9 [0.5-1.48]	0.4 [0.3-0.5]	0.005	0.7 [0.47-1.52]	0.53 [0.4-0.7]	0.058§
Bicarbonate, mmol/L, Median [IQR]	day 1	21.5 [20.5-22.33]	20.0 [20.0-20.0]	0.044	22.0 [20.08-24.75]	21.0 [19.0-23.33]	0.566	17.5 [16.65-19.71]	20.33 [16.25-22.5]	0.191
	day 3	20.0 [19.0-23.75]	25.0 [24.0-26.5]	0.176	25.5 [23.0-27.75]	24.0 [24.0-29.0]	0.873	19.0 [16.58-20.75]	21.0 [19.5-23.5]	0.011
CK, U/L, Median [IQR]	day 1	184.0 [91.61-5574.75]	816.0 [542.0-956.5]	0.247	276.36 [185.38-397.21]	100.0 [74.0-1140.0]	0.081	230.0 [184.0-503.25]	157.5 [81.3-461.5]	0.072
	day 3	184.0 [47.22-5574.75]	816.0 [479.5-2702.0]	0.324	226.5 [148.5-454.31]	110.33 [46.0-1140.0]	0.221	184.0 [167.0-530.25]	193.5 [94.25-440.75]	0.223
Creatinine, mg/dL, Median [IQR]	day 1	0.7 [0.65-1.05]	1.3 [1.15-1.35]	0.104	0.95 [0.8-1.37]	0.8 [0.7-1.1]	0.186	2.65 [2.08-2.95]	2.0 [1.4-2.55]	0.043
	day 3	1.2 [0.67-4.0]	0.92 [0.81-1.15]	0.454	1.2 [0.8-2.18]	0.9 [0.7-1.1]	0.075	4.35 [3.5-5.37]	2.6 [1.25-4.45]	0.037
CRP, mg/dL, Median [IQR]	day 1	15.5 [11.21-16.48]	17.64 [17.25-21.73]	0.571	19.38 [15.27-23.98]	15.89 [12.9-25.77]	0.329	18.28 [15.71-21.94]	19.4 [14.9-24.14]	0.398
	day 3	15.5 [7.46-16.38]	14.0 [13.35-15.43]	0.996	22.34 [13.66-28.63]	15.5 [8.95-25.54]	0.154	20.46 [16.92-26.57]	16.6 [12.32-22.08]	0.160
D-dimer, ng/mL, Median [IQR]	day 1	1508.0 [870.0-1508.0]	947.5 [643.75-1227.75]	0.473	1208.12 [631.75-2086.08]	1660.0 [1071.0-2520.08]	0.201	1627.5 [1508.0-3461.25]	1792.0 [1125.5-3857.5]	0.380
	day 3	1508.0 [582.5-1508.0]	1077.0 [763.5-1292.5]	0.362	1508.0 [1005.62-3546.25]	1833.0 [1071.0-3152.0]	0.399	1582.5 [1508.0-2959.17]	1630.0 [1012.5-4950.0]	0.434

ESR, mm/hr, Median [IQR]	day 1	88.0 [82.5-88.0]	88.0 [79.67-112.5]	0.350	88.0 [72.0-90.0]	93.0 [87.0-102.0]	0.132	87.33 [62.0-88.0]	96.0 [67.5-111.88]	0.149
	day 3	88.0 [84.5-88.0]	88.0 [86.5-102.1]	0.260	88.0 [80.05-95.42]	93.0 [87.0-102.0]	0.191	88.0 [74.7-89.2]	100.0 [65.0-110.0]	0.176
Ferritin, ng/mL, Median [IQR]	day 1	1099.24 [1099.24-1981.2]	1562.54 [862.0-3349.78]	0.500	1562.43 [1158.5-2129.01]	1099.24 [521.71-1663.19]	0.043	1136.39 [637.23-1349.16]	1194.6 [798.74-1700.12]	0.287
	day 3	1099.24 [1099.24-1895.6]	1169.2 [665.33-2176.3]	0.500	1446.26 [1156.0-2133.35]	1062.76 [433.13-1357.1]	0.015	1098.62 [575.57-1332.58]	1099.24 [759.14-1641.97]	0.276
Globulin, g/dL, Median [IQR]	day 1	3.0 [2.5-3.0]	3.0 [2.5-3.12]	0.852	3.0 [3.0-3.48]	3.0 [3.0-3.5]	0.500	3.0 [2.88-3.62]	3.0 [3.0-3.5]	0.456
	day 3	3.0 [2.0-3.0]	3.3 [2.65-3.55]	0.202	3.0 [3.0-3.31]	3.0 [3.0-3.0]	0.123	3.0 [2.75-3.35]	3.0 [3.0-3.34]	0.462
Glucose, mg/dL, Median [IQR]	day 1	151.0 [135.5-177.5]	127.25 [116.88-165.38]	0.653	117.25 [100.25-160.0]	141.0 [107.0-178.33]	0.100	189.38 [153.25-234.81]	171.0 [120.33-212.75]	0.169
	day 3	151.0 [126.5-335.5]	171.0 [168.5-195.0]	0.410	159.75 [135.38-191.12]	169.33 [115.0-238.0]	0.480	205.0 [155.79-290.38]	200.0 [141.5-257.17]	0.577
Hemoglobin, g/dL, Median [IQR]	day 1	12.4 [12.05-13.0]	12.85 [11.88-13.19]	0.941	12.23 [11.45-13.28]	11.7 [10.6-12.0]	0.067	12.23 [11.66-13.44]	12.27 [10.3-13.68]	0.365
	day 3	12.4 [10.5-13.85]	10.9 [10.55-12.0]	0.747	11.17 [10.55-12.15]	10.28 [9.1-11.2]	0.031[§]	10.65 [10.04-12.01]	10.8 [9.4-11.6]	0.611
LDH, U/L, Median [IQR]	day 1	542.0 [499.25-624.21]	622.0 [451.0-738.0]	0.935	538.7 [452.38-675.75]	438.0 [348.5-508.5]	0.039	639.0 [472.5-804.0]	464.12 [424.7-780.25]	0.103
	day 3	524.0 [476.75-568.43]	442.0 [361.0-650.5]	0.903	503.38 [467.5-649.5]	411.67 [371.0-495.5]	0.010	560.0 [472.5-615.75]	459.0 [402.5-658.75]	0.109
Lactic acid level, mmol/L, Mean [CI]	day 1	3.35 [0.83,5.88]	2.02 [0.86,3.18]	0.439	1.96 [1.36,2.55]	2.1 [1.28,2.91]	0.386	2.81 [1.9,3.72]	1.89 [1.43,2.36]	0.012
	day 3	3.49 [0.82,6.17]	1.82 [0.27,3.37]	0.410	1.95 [1.38,2.53]	1.82 [1.24,2.4]	0.399	2.62 [1.54,3.69]	1.63 [1.23,2.03]	0.036
Lymphocyte percentage, %, Median [IQR]	day 1	8.9 [5.95-14.35]	4.4 [3.5-6.4]	0.177	9.85 [7.28-15.05]	9.0 [7.7-12.3]	0.480	7.6 [4.46-13.21]	5.9 [3.56-9.19]	0.165
	day 3	7.5 [6.35-10.95]	10.85 [9.03-11.68]	0.412	8.5 [5.45-10.76]	9.9 [6.5-12.3]	0.275	6.12 [3.27-7.58]	7.23 [4.13-10.1]	0.611
Lymphocyte count, x10(9)/L, Median [IQR]	day 1	0.73 [0.63-0.89]	0.65 [0.43-0.78]	0.247	0.86 [0.67-1.17]	0.75 [0.52-1.29]	0.322	0.82 [0.58-1.12]	0.63 [0.44-0.87]	0.190
	day 3	0.98 [0.62-1.13]	0.73 [0.64-0.84]	0.505	0.77 [0.64-0.98]	0.63 [0.59-0.99]	0.335	0.7 [0.53-0.82]	0.65 [0.42-0.82]	0.363
Neutrophil percentage, %, Median [IQR]	day 1	84.3 [79.95-90.6]	87.9 [87.65-89.52]	0.353	85.15 [77.92-86.62]	85.63 [80.6-87.7]	0.386	87.55 [82.26-92.25]	90.2 [83.6-91.85]	0.189
	day 3	86.2 [85.02-89.05]	83.45 [81.33-85.78]	0.235	84.45 [79.8-86.38]	84.25 [78.3-90.0]	0.382	88.5 [82.61-92.5]	86.1 [82.8-90.73]	0.800
Neutrophil count, x10(9)/L, Median [IQR]	day 1	8.67 [6.33-10.99]	9.49 [8.24-11.52]	0.684	7.71 [5.64-9.38]	8.0 [5.85-10.05]	0.568	10.11 [7.11-11.52]	10.37 [6.73-13.11]	0.927

	day 3	9.7 [7.73-11.6]	7.67 [5.58-8.33]	0.158	8.35 [7.24-9.63]	6.79 [5.15-10.05]	0.497	9.94 [9.19-13.09]	8.97 [6.9-11.21]	0.145
Platelet, x10(9)/L, Median [IQR]	day 1	200.0 [188.5-299.5]	205.0 [179.0-254.0]	0.596	231.0 [172.75-277.75]	313.0 [295.0-371.0]	0.012§	202.5 [143.5-266.25]	235.0 [205.5-303.0]	0.090
	day 3	262.0 [192.0-282.0]	258.0 [197.5-289.0]	0.950	246.5 [164.25-314.5]	308.0 [223.0-368.0]	0.108	193.5 [163.75-235.75]	224.0 [175.5-310.0]	0.146
Potassium, mmol/L, Median [IQR]	day 1	4.1 [3.8-4.47]	4.55 [4.53-4.71]	0.214	3.98 [3.6-4.3]	4.0 [3.8-4.4]	0.300	4.47 [3.86-5.26]	4.2 [3.98-4.8]	0.386
	day 3	3.95 [3.9-4.45]	4.7 [4.15-4.9]	0.362	4.2 [3.82-4.6]	4.1 [4.1-4.45]	0.989	4.75 [4.1-5.04]	4.15 [3.87-4.6]	0.055
Procalcitonin, ng/mL, Median [IQR]	day 1	0.42 [0.1-0.68]	0.3 [0.3-0.45]	0.500	0.89 [0.2-2.14]	0.36 [0.18-0.95]	0.126	1.61 [0.59-3.14]	0.98 [0.42-1.48]	0.133
	day 3	0.53 [0.28-8.92]	0.22 [0.2-0.25]	0.181	1.17 [0.38-3.14]	0.44 [0.18-1.5]	0.078	2.69 [1.11-4.94]	1.15 [0.51-1.83]	0.016§
Sodium, mmol/L, Median [IQR]	day 1	137.0 [135.5-141.0]	142.0 [141.88-142.5]	0.207	139.33 [134.25-142.38]	138.0 [134.0-141.0]	0.188	140.0 [136.25-142.25]	138.0 [135.25-139.75]	0.083
	day 3	140.0 [135.75-142.75]	145.0 [142.5-147.5]	0.136	142.25 [139.0-145.88]	141.0 [137.0-142.0]	0.068	140.5 [137.62-142.75]	138.0 [136.5-141.5]	0.463
Troponin, ng/mL, Mean [CI]	day 1	0.06 [0.01,0.11]	0.0 [0.0,0.0]	0.071	0.43 [-0.04,0.9]	0.11 [0.03,0.19]	0.387	0.26 [0.09,0.44]	0.5 [-0.04,1.05]	0.054
	day 3	0.06 [0.01,0.11]	0.01 [-0.03,0.05]	0.157	0.53 [-0.1,1.16]	0.09 [0.03,0.15]	0.443	0.34 [0.1,0.57]	0.54 [-0.0,1.08]	0.118
Triglycerides, mg/dL, Median [IQR]	day 1	228.0 [228.0-274.5]	283.56 [202.78-424.78]	0.409	227.0 [163.25-321.0]	228.0 [149.0-238.5]	0.425	258.0 [154.5-311.25]	203.0 [150.0-273.5]	0.206
	day 3	228.0 [228.0-303.75]	311.0 [216.5-319.83]	0.745	213.0 [144.5-322.4]	228.0 [149.0-359.0]	0.386	258.0 [154.25-309.0]	203.0 [154.5-268.5]	0.219
WBC, x10(9)/L, Median [IQR]	day 1	7.8 [6.07-12.1]	10.8 [9.4-12.84]	0.471	9.0 [6.8-10.97]	12.1 [9.1-15.63]	0.026§	11.78 [10.49-13.62]	13.1 [8.15-14.57]	0.652
	day 3	12.1 [10.68-14.3]	10.0 [7.2-10.1]	0.104	10.05 [8.43-12.07]	8.5 [7.9-11.0]	0.529	11.5 [11.03-15.11]	9.7 [8.05-12.25]	0.088
GCS, Mean [CI]	day 1	8.71 [4.49,12.94]	5.67 [-0.07,11.4]	0.204	5.91 [5.09,6.73]	4.85 [3.75,5.95]	0.060	5.25 [4.07,6.43]	4.3 [3.43,5.17]	0.048
	day 3	4.57 [2.45,6.7]	7.67 [-4.84,20.17]	0.165	5.5 [4.57,6.43]	6.15 [4.74,7.57]	0.122	3.17 [2.92,3.41]	5.04 [4.1,5.97]	0.013§
MAP, mmHg, Median [IQR]	day 1	84.29 [80.99-91.58]	79.3 [76.53-86.33]	0.583	82.91 [76.51-85.4]	81.05 [77.78-86.6]	0.892	80.88 [78.56-86.04]	85.46 [79.92-89.46]	0.173
	day 3	84.05 [82.19-88.57]	78.75 [77.45-96.54]	0.713	80.45 [79.41-84.98]	78.77 [75.79-81.52]	0.090	80.21 [76.8-86.76]	83.44 [79.5-88.5]	0.147
Temperature, degrees C, Median [IQR]	day 1	37.04 [36.52-37.56]	37.22 [36.77-37.6]	0.894	37.47 [37.13-38.01]	37.23 [36.95-37.47]	0.193	36.99 [36.7-37.47]	37.05 [36.63-37.35]	0.968

	day 3	37.65 [36.96-37.83]	36.9 [36.76-37.17]	0.322	37.52 [37.03-37.94]	37.25 [37.02-37.4]	0.251	36.73 [36.47-37.2]	37.0 [36.43-37.29]	0.718
Urine output, mL, Median [IQR]	day 1	1295.0 [1126.25-1575.0]	1160.0 [1040.0-1432.5]	0.693	1270.0 [878.75-1610.0]	1005.0 [895.0-1160.0]	0.216	647.5 [414.0-1112.25]	491.0 [260.0-777.5]	0.233
	day 3	800.0 [716.5-1048.75]	1095.0 [1072.5-1647.5]	0.127	1212.5 [878.75-1828.75]	1525.0 [885.0-1780.0]	0.983	587.0 [151.25-728.75]	875.0 [197.5-1590.0]	0.062
FiO2, %, Median [IQR]	day 1	73.64 [63.33-87.95]	58.0 [53.72-72.85]	0.370	75.15 [66.47-87.81]	75.0 [59.0-90.0]	0.928	73.98 [68.7-94.44]	82.5 [76.42-87.39]	0.595
	day 3	63.85 [53.79-77.5]	44.76 [42.38-47.23]	0.117	59.58 [50.21-69.72]	47.5 [40.0-60.36]	0.015§	55.59 [50.0-61.25]	50.0 [40.0-63.5]	0.330
PaO2, mmHg, Median [IQR]	day 1	105.17 [90.54-120.18]	116.5 [110.72-216.73]	0.141	92.13 [85.98-116.69]	112.15 [100.8-154.0]	0.049	111.68 [89.31-121.4]	121.6 [98.97-150.13]	0.074
	day 3	85.6 [80.85-107.67]	88.0 [82.53-93.33]	0.789	78.35 [71.7-96.82]	91.1 [81.1-106.17]	0.165	92.12 [75.71-98.27]	95.65 [75.89-108.75]	0.219
P/F ratio, Median [IQR]	day 1	145.87 [142.13-151.72]	235.62 [208.28-298.53]	0.009§	128.76 [104.16-165.83]	151.33 [116.33-201.29]	0.183	147.07 [105.41-186.95]	141.9 [120.55-192.97]	0.319
	day 3	148.64 [107.08-174.8]	192.63 [184.83-206.53]	0.143	142.17 [112.21-171.39]	212.35 [147.37-248.25]	0.021	156.31 [126.46-205.74]	178.2 [132.6-278.98]	0.140
SpO2, Median [IQR]	day 1	95.65 [92.65-97.23]	96.95 [95.59-97.74]	0.526	95.77 [94.45-97.0]	97.76 [96.43-98.83]	0.010	96.48 [95.87-97.66]	97.21 [95.26-98.0]	0.894
	day 3	92.83 [92.07-97.14]	96.79 [95.63-97.3]	0.354	94.98 [92.94-95.96]	95.42 [94.04-96.18]	0.144	96.43 [94.91-97.98]	96.29 [95.32-97.83]	0.986
Driving pressure, Median [IQR]	day 1	13.5 [11.5-16.66]	10.71 [9.86-12.86]	0.271	12.22 [7.94-14.0]	14.14 [12.0-16.0]	0.045	12.39 [7.55-13.5]	12.0 [10.35-14.53]	0.239
	day 3	12.5 [10.67-14.25]	11.08 [10.54-12.38]	0.803	12.6 [10.44-14.89]	14.0 [12.0-16.0]	0.372	11.0 [10.57-14.12]	13.0 [12.0-13.55]	0.161
Minute ventilation, L, Median [IQR]	day 1	9.4 [7.87-10.53]	9.65 [9.35-10.6]	0.467	8.8 [7.93-10.46]	9.81 [8.75-11.23]	0.113	9.45 [8.03-10.32]	9.18 [8.07-10.83]	0.976
	day 3	10.05 [8.15-10.18]	9.56 [9.34-10.43]	0.410	9.08 [7.86-10.62]	8.5 [7.83-10.2]	0.231	11.45 [9.66-14.26]	9.8 [8.99-10.44]	0.039
PCO2, mmHg, Median [IQR]	day 1	37.97 [33.99-42.33]	41.95 [37.55-45.48]	0.496	42.02 [37.95-46.2]	41.48 [39.3-45.82]	0.356	45.47 [43.3-48.87]	41.45 [38.37-47.85]	0.564
	day 3	43.57 [41.22-44.42]	39.0 [34.5-41.95]	0.153	47.88 [40.14-55.46]	41.3 [39.4-50.75]	0.121	41.18 [36.33-45.3]	40.88 [36.72-44.1]	0.936
PEEP, Median [IQR]	day 1	10.0 [7.59-11.86]	11.29 [10.64-12.89]	0.310	11.83 [10.0-12.63]	12.0 [9.0-14.17]	0.844	12.14 [9.93-14.95]	11.62 [9.39-13.17]	0.240
	day 3	11.33 [10.0-12.75]	9.21 [7.77-9.27]	0.119	12.0 [10.12-13.78]	10.33 [9.2-14.0]	0.864	12.0 [11.88-13.85]	10.8 [9.7-13.1]	0.084
PH, Mean [CI]	day 1	7.38 [7.33,7.42]	7.35 [7.19,7.52]	0.542	7.36 [7.34,7.39]	7.39 [7.36,7.43]	0.151	7.29 [7.25,7.32]	7.32 [7.29,7.35]	0.157

	day 3	7.33 [7.26,7.4]	7.35 [7.14,7.56]	0.688	7.36 [7.33,7.39]	7.4 [7.37,7.43]	0.084	7.29 [7.26,7.33]	7.35 [7.33,7.37]	0.002§
PIP, Median [IQR]	day 1	27.62 [25.33-28.83]	26.0 [23.6-27.0]	0.284	28.23 [23.96-31.3]	28.0 [26.67-32.25]	0.329	28.75 [25.32-31.8]	26.5 [24.67-30.83]	0.287
	day 3	29.5 [29.08-32.12]	20.67 [20.58-22.67]	0.014§	28.88 [26.15-30.5]	28.8 [26.5-32.75]	0.253	28.42 [26.96-33.25]	26.0 [24.0-30.0]	0.066
Plateau pressure, Median [IQR]	day 1	25.0 [24.58-27.58]	22.0 [20.5-23.5]	0.061	22.88 [18.0-27.0]	25.5 [24.0-28.0]	0.200	24.25 [18.92-25.62]	24.0 [17.88-25.75]	0.964
	day 3	25.0 [21.5-27.75]	20.44 [18.93-21.72]	0.273	25.38 [22.19-27.88]	26.0 [23.0-27.0]	0.802	25.0 [23.75-27.62]	24.0 [21.75-25.83]	0.237
Static compliance, Median [IQR]	day 1	28.0 [25.25-30.35]	37.33 [33.48-38.11]	0.135	28.13 [22.61-33.64]	24.78 [22.64-27.93]	0.100	32.71 [25.69-40.56]	29.11 [21.08-35.47]	0.121
	day 3	30.0 [22.95-34.44]	31.0 [30.86-33.9]	0.247	27.8 [23.54-31.61]	25.71 [21.88-27.22]	0.226	34.59 [30.36-38.28]	30.75 [26.7-35.41]	0.147
Tidal PBW ratio, Median [IQR]	day 1	6.8 [6.6-7.39]	6.04 [6.0-6.16]	0.174	6.52 [6.28-7.31]	6.68 [6.32-8.25]	0.217	6.02 [5.75-6.63]	6.5 [6.1-6.6]	0.240
	day 3	6.49 [5.87-6.66]	6.16 [6.06-6.37]	0.951	6.5 [6.01-6.95]	6.52 [6.29-6.68]	0.399	6.05 [5.82-6.73]	6.52 [6.24-6.8]	0.150
Tidal volume, mL, Median [IQR]	day 1	408.33 [390.0-435.0]	400.0 [375.0-400.0]	0.148	413.0 [370.0-450.0]	395.0 [380.0-410.0]	0.309	400.0 [350.0-435.0]	400.0 [350.0-420.0]	0.890
	day 3	380.0 [345.0-390.0]	407.78 [378.89-413.89]	0.485	400.0 [362.5-442.5]	373.33 [350.0-400.0]	0.256	420.0 [372.5-444.38]	400.0 [366.25-420.0]	0.745
Ventilator ratio, Median [IQR]	day 1	1.69 [1.31-1.9]	1.72 [1.51-2.04]	0.584	1.86 [1.53-1.99]	1.96 [1.71-2.04]	0.235	1.75 [1.59-2.01]	1.86 [1.76-2.02]	0.385
	day 3	1.77 [1.63-2.37]	1.84 [1.59-1.85]	0.411	2.04 [1.67-2.2]	1.85 [1.52-1.99]	0.269	2.01 [1.87-2.2]	1.89 [1.68-1.99]	0.402

† p-value calculated by student's t-test/Mann-Whitney test
** False discovery rate corrected p-value < 0.05
§ Age adjusted (analysis of covariance [ANCOVA]) p-value < 0.05
ALT=Alanine aminotransferase, AST=Aspartate aminotransferase, CI=confidence interval, CK=Creatine kinase, CRP=C-reactive protein, ESR=Erythrocyte sedimentation rate, FiO2=fraction of inspired oxygen, GCS=Glasgow Coma Scale, IQR=Interquartile range, LDH=Lactate dehydrogenase, MAP=Mean arterial pressure, PaO2=partial pressure of oxygen, PBW=predicted body weight, PCO2=arterial partial pressure of carbon dioxide, PEEP=Positive end-expiratory pressure, PIP=Peak inspiratory pressure, P/F ratio=PaO2/FiO2 ratio, SpO2=oxygen saturation.

Figures

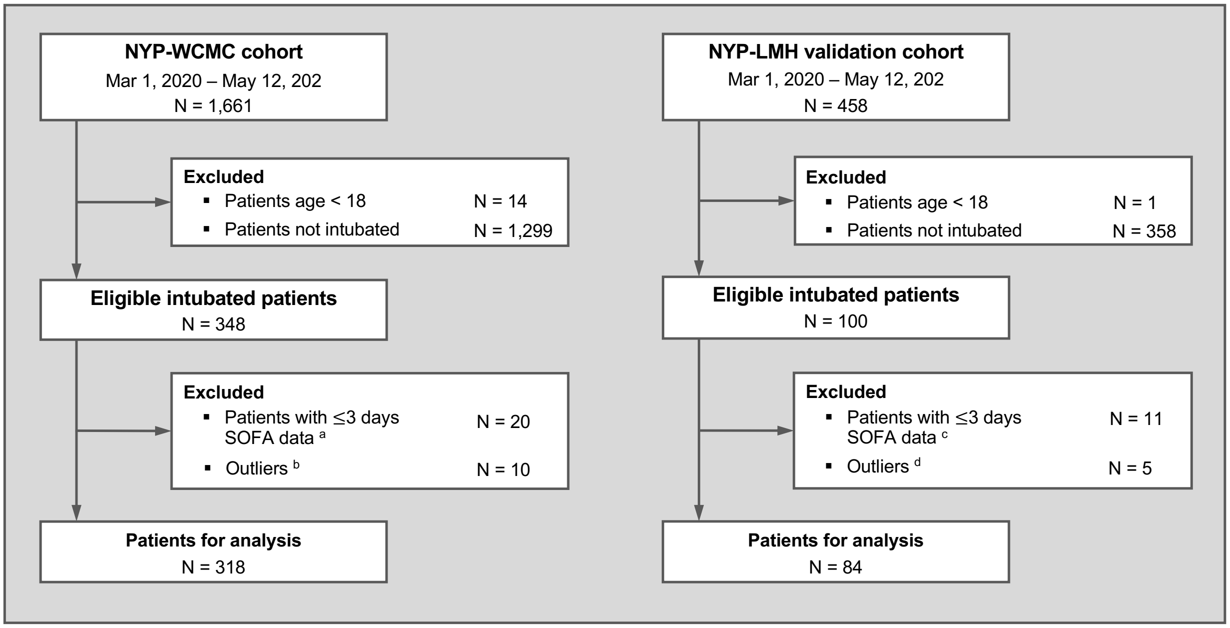

Figure S1. Patient exclusion criteria. Abbreviations: SOFA=Sequential Organ Failure Assessment.

[a] Out of the 20 patients, 9, 3, and 3 dead within day 1, 2, 3 after intubation, respectively; 2 have no records, 2 and 1 only have 2- and 3-days SOFA data, respectively.

[b] Out of the 10 patients, 7 have no change of SOFA score within 7 days after intubation, 3 whose SOFA trajectories fluctuated heavily.

[c] Out of the 11 patients, 2, 5, and 4 dead within day 1, 2, 3 after intubation, respectively.

[d] SOFA trajectories of the 5 patients fluctuated heavily.

Abbreviation: NYP-LMH=New York Presbyterian Hospital-Lower Manhattan Hospital, SD=standard deviation, SOFA=Sequential Organ Failure Assessment

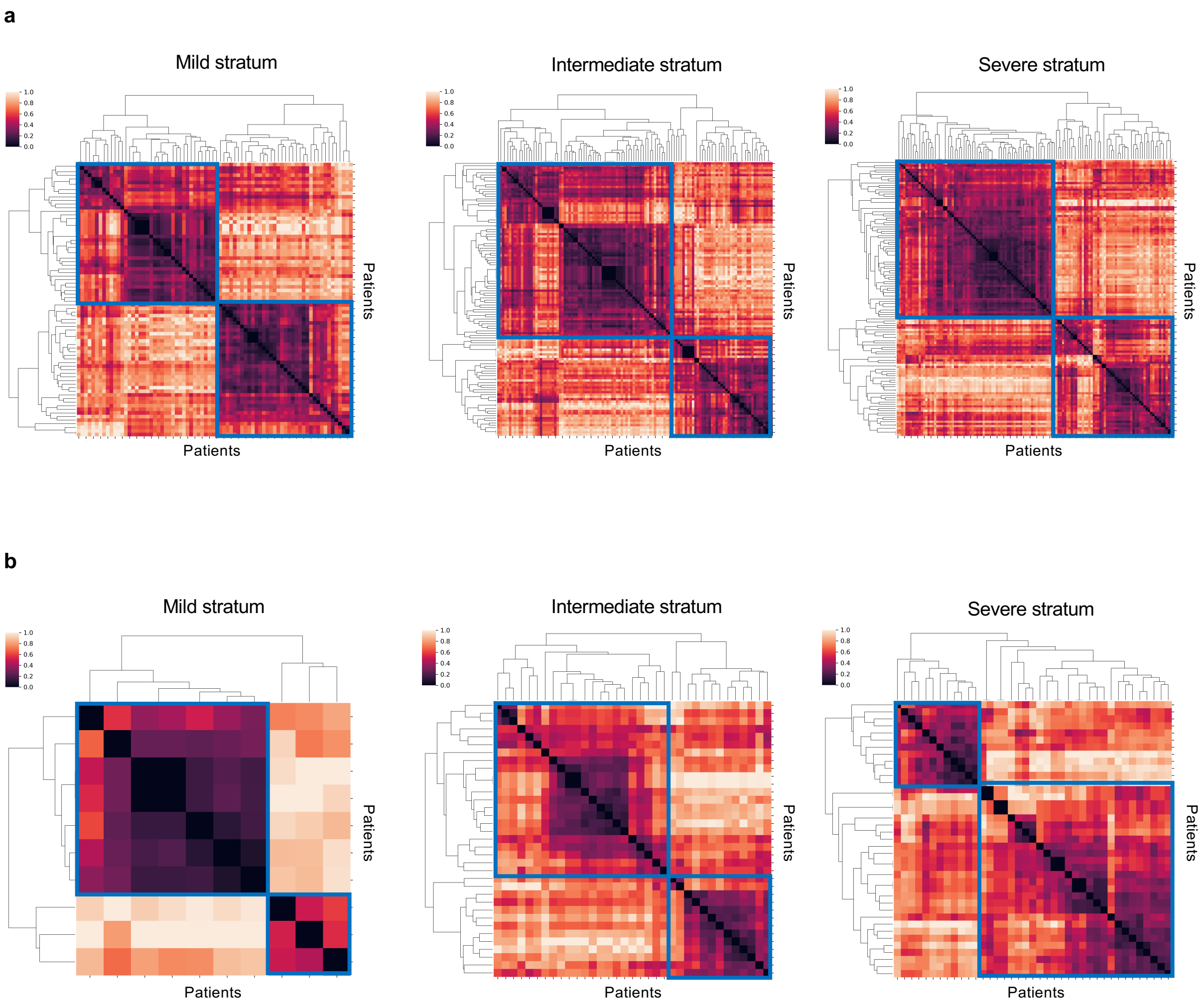

Figure S2. Clustergrams of hierarchical clustering. Horizontal and vertical axes represent patients. Color intensity denotes normalized pairwise patient similarity derived using Dynamic Time Warping (DTW). All clustergrams suggest optimal cluster number 2. (**a**) Clustergrams derived from the NYP-WCMC cohort. (**b**) Clustergrams derived from the NYP-LMH validation cohort.

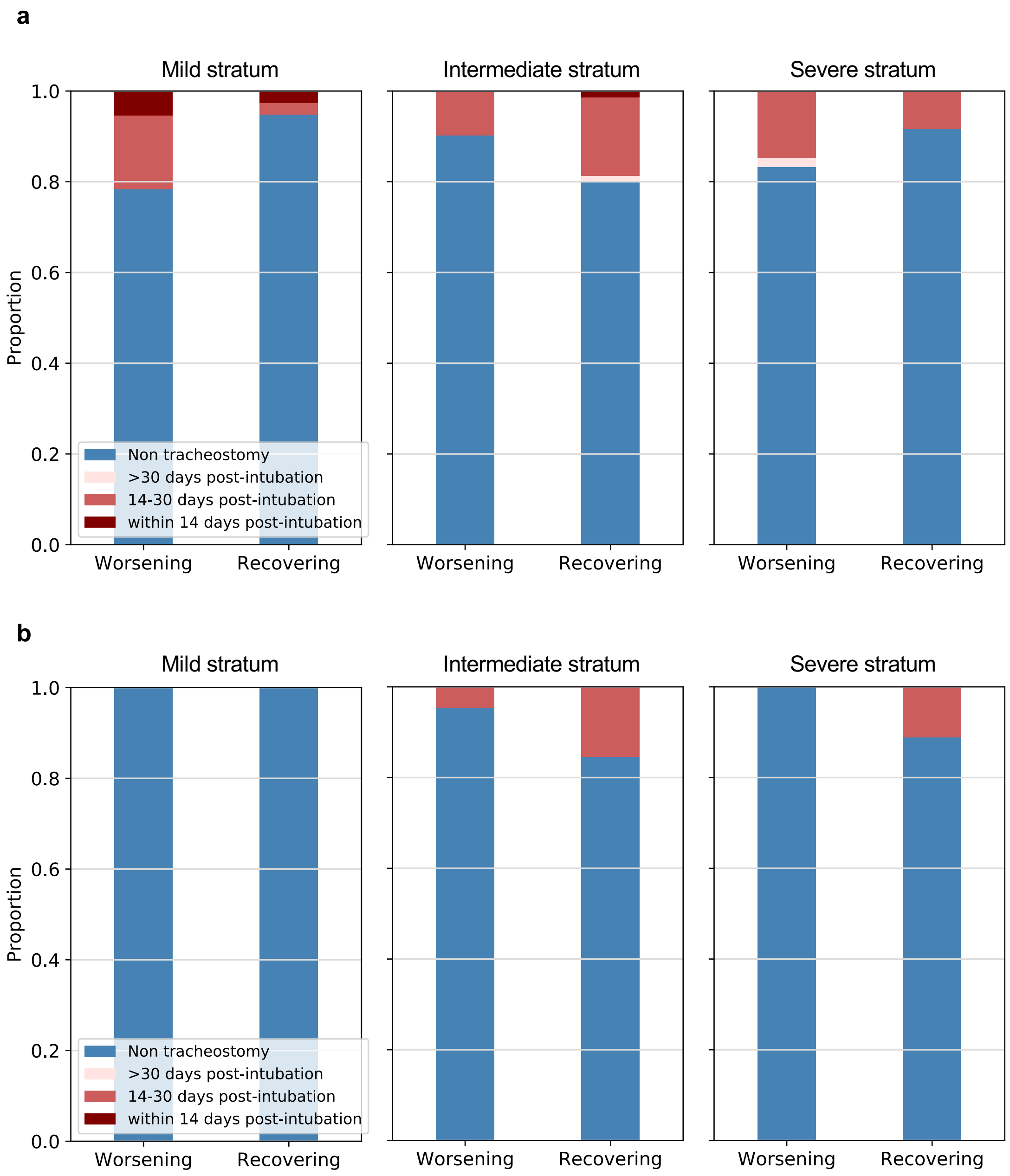

Figure S3. Tracheostomy outcome of the trajectory subphenotypes. (**a**) Statistics of tracheostomy of subphenotypes within the NYP-WCM cohort; (**b**) Statistics of tracheostomy of subphenotypes within the NYP-LMH validation cohort.

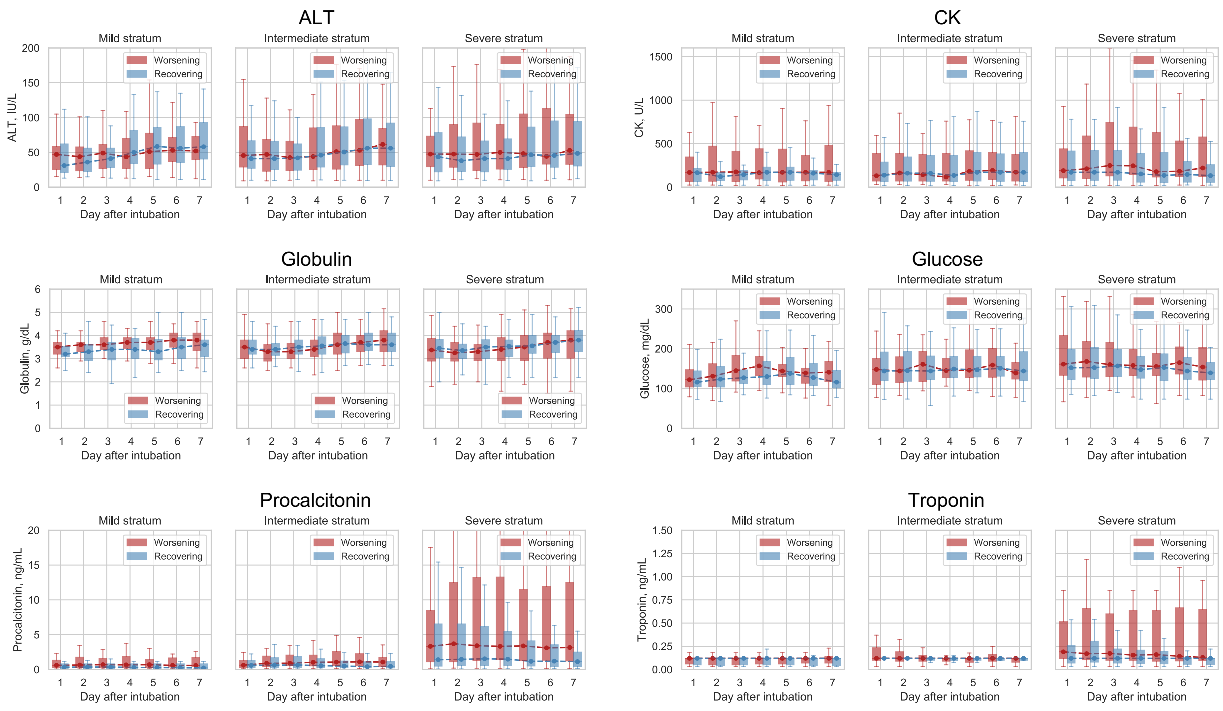

Figure S4. Laboratory test value trajectories of the identified subphenotypes.

Abbreviations: ALT=Alanine aminotransferase, CK=Creatine kinase.

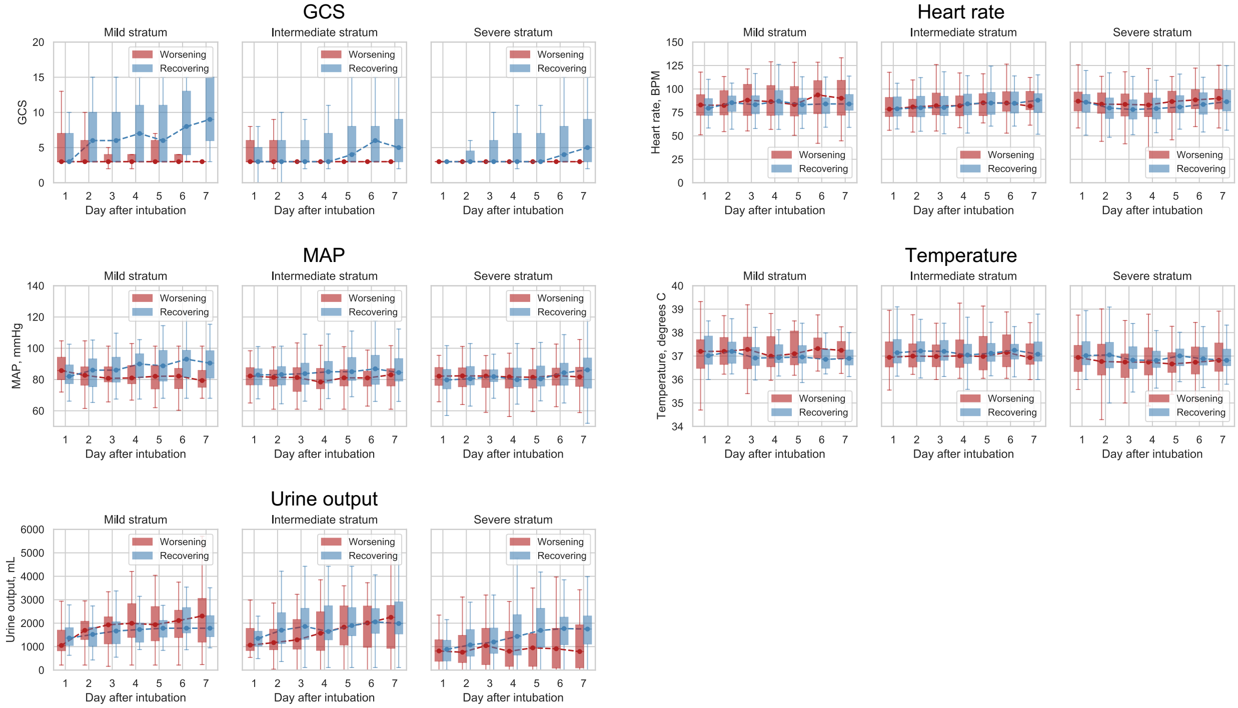

Figure S5. Vital sign trajectories of the identified subphenotypes.

Abbreviations: GCS=Glasgow Coma Scale, MAP=Mean arterial pressure.

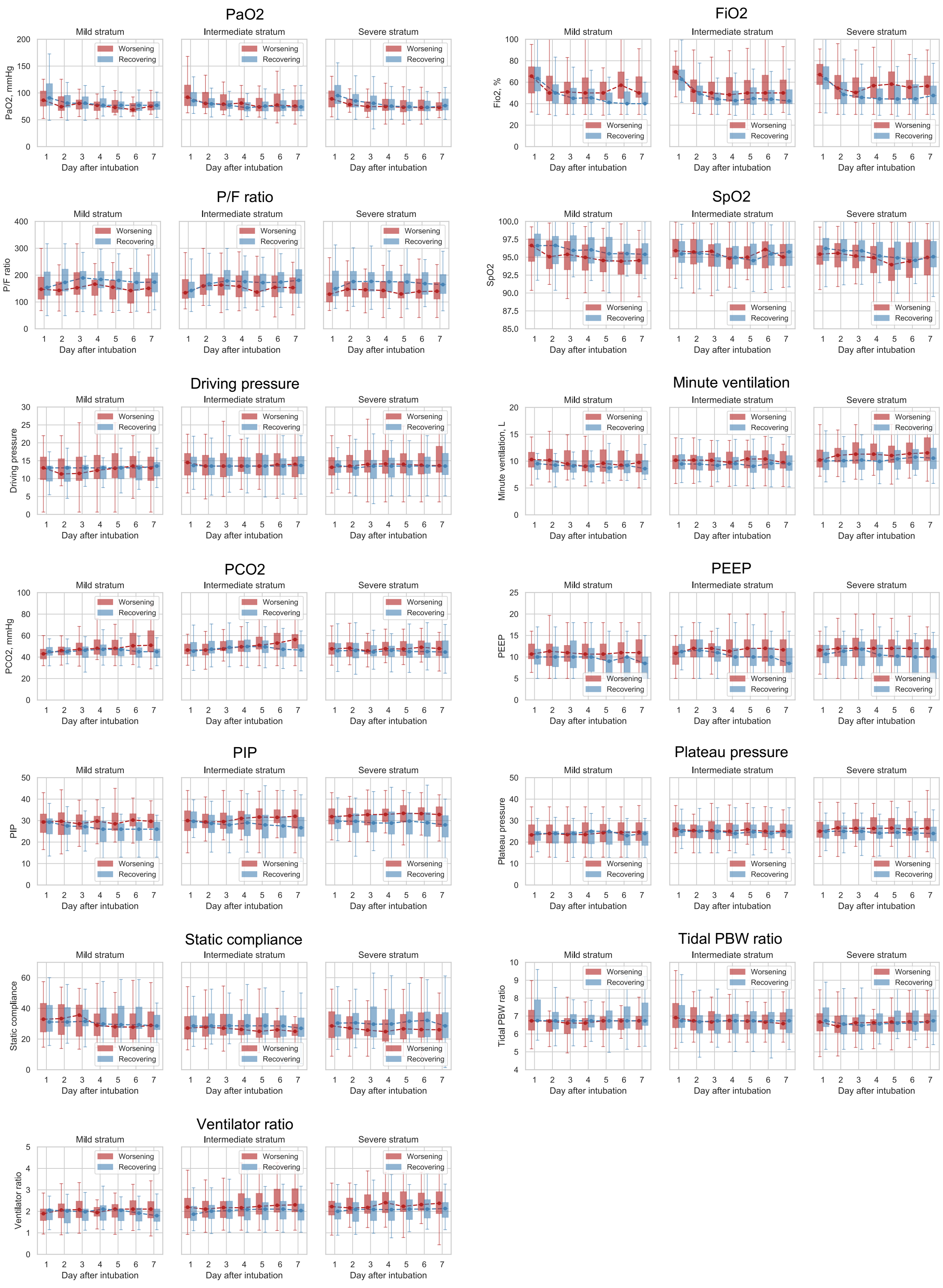
PaO2
FiO2
P/F ratio
SpO2
Driving pressure
Minute ventilation
PCO2
PEEP
PIP
Plateau pressure
Static compliance
Tidal PBW ratio
Ventilator ratio
Mild stratum
Intermediate stratum
Severe stratum
Worsening
Recovering
Day after intubation
PaO2, mmHg
Fio2, %
P/F ratio
SpO2
Driving pressure
Minute ventilation, L
PCO2, mmHg
PEEP
PIP
Plateau pressure
Static compliance
Tidal PBW ratio
Ventilator ratio

Figure S6. Respiratory variables trajectories of the identified subphenotypes.

Abbreviations: PaO2=partial pressure of oxygen, PBW=predicted body weight, PCO2=arterial partial pressure of carbon dioxide, PEEP=Positive end-expiratory pressure, PIP=Peak inspiratory pressure, P/F ratio=PaO2/FiO2 ratio, SpO2=oxygen saturation.

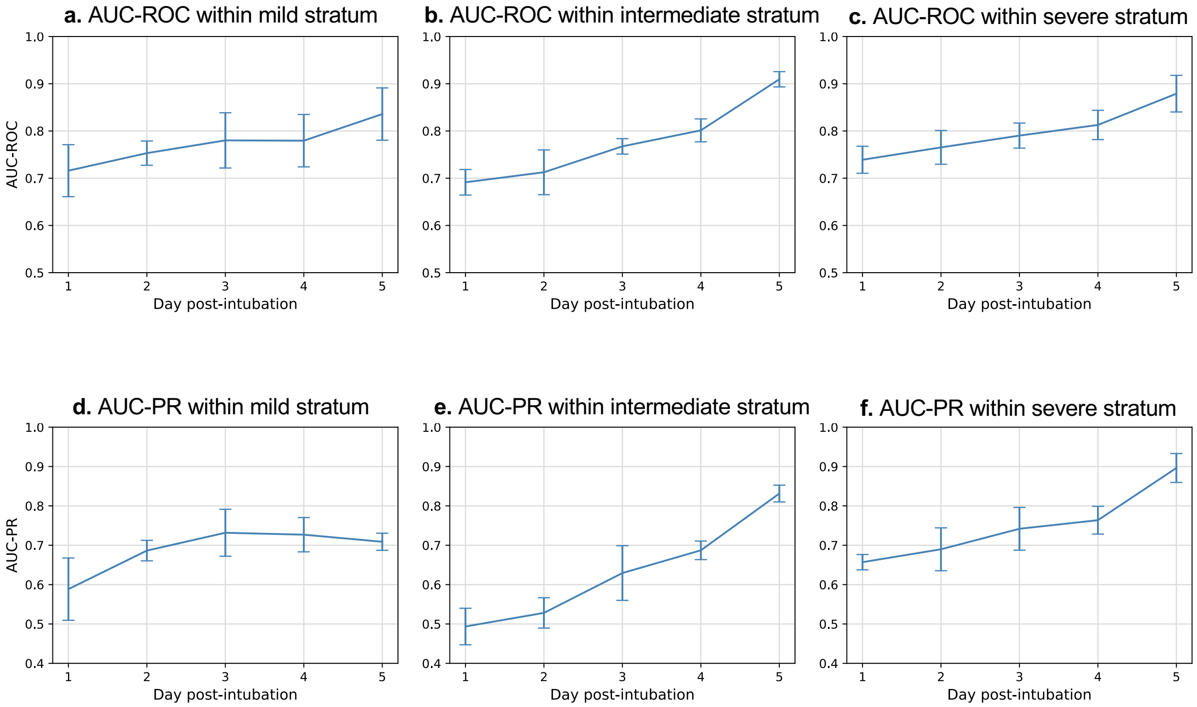

Figure S7. Prediction performances of subphenotype prediction models within baseline mild, intermediate, and severe strata, respectively. (**a-c**) Prediction performances in terms of AUC-ROC scores. An AUC-ROC measures accuracy of a prediction model by comprehensively considering true positive rate and false positive rate in prediction. (**d-f**) Prediction performances in terms of AUC-PR scores.

Abbreviations: AUC-PR=Area Under the Precision Recall Curve; AUC-ROC=Area Under the Receiver Operating Characteristics.

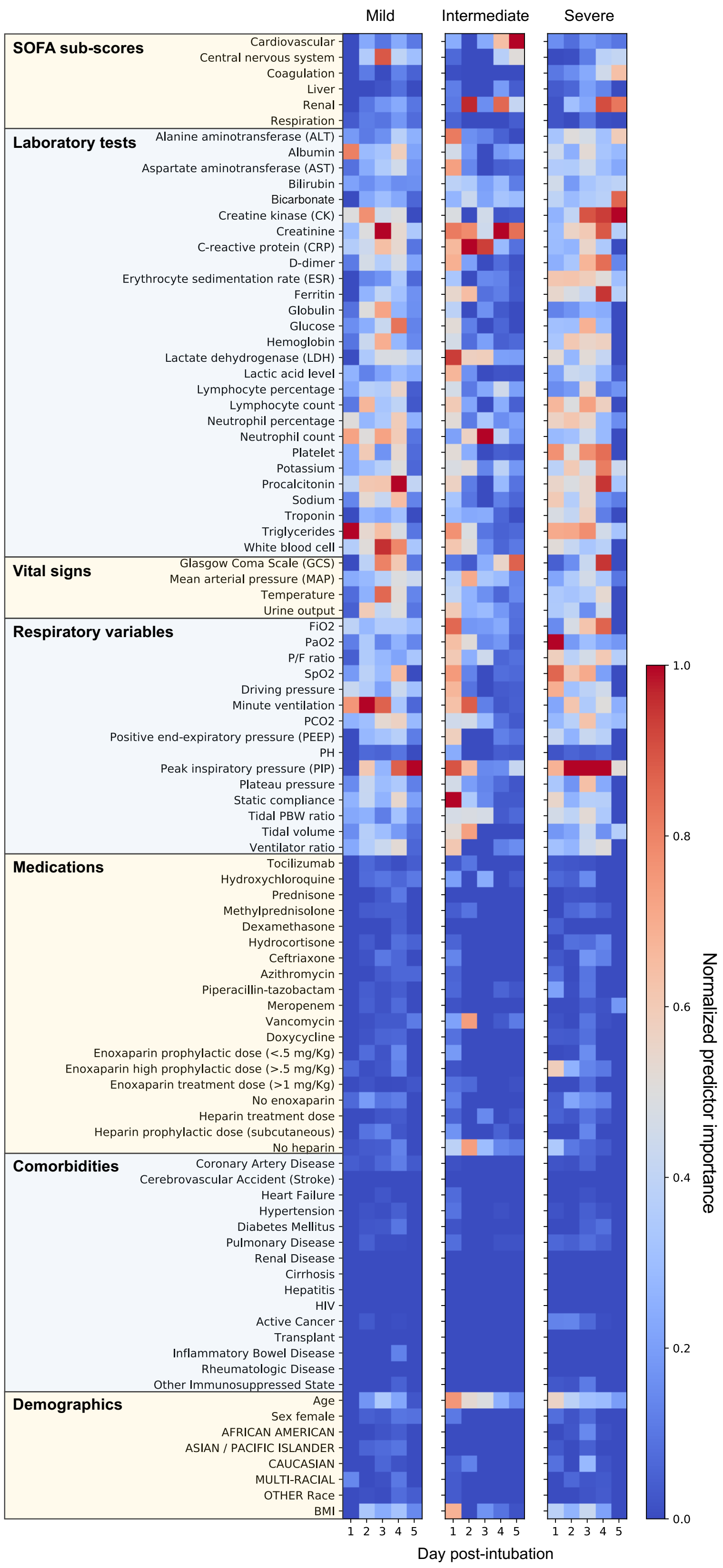
Mild
Intermediate
Severe
SOFA sub-scores
Cardiovascular
Central nervous system
Coagulation
Liver
Renal
Respiration
Laboratory tests
Alanine aminotransferase (ALT)
Albumin
Aspartate aminotransferase (AST)
Bilirubin
Bicarbonate
Creatine kinase (CK)
Creatinine
C-reactive protein (CRP)
D-dimer
Erythrocyte sedimentation rate (ESR)
Ferritin
Globulin
Glucose
Hemoglobin
Lactate dehydrogenase (LDH)
Lactic acid level
Lymphocyte percentage
Lymphocyte count
Neutrophil percentage
Neutrophil count
Platelet
Potassium
Procalcitonin
Sodium
Troponin
Triglycerides
White blood cell
Vital signs
Glasgow Coma Scale (GCS)
Mean arterial pressure (MAP)
Temperature
Urine output
Respiratory variables
FiO2
PaO2
P/F ratio
SpO2
Driving pressure
Minute ventilation
PCO2
Positive end-expiratory pressure (PEEP)
PH
Peak inspiratory pressure (PIP)
Plateau pressure
Static compliance
Tidal PBW ratio
Tidal volume
Ventilator ratio
Medications
Tocilizumab
Hydroxychloroquine
Prednisone
Methylprednisolone
Dexamethasone
Hydrocortisone
Ceftriaxone
Azithromycin
Piperacillin-tazobactam
Meropenem
Vancomycin
Doxycycline
Enoxaparin prophylactic dose (<.5 mg/Kg)
Enoxaparin high prophylactic dose (>.5 mg/Kg)
Enoxaparin treatment dose (>1 mg/Kg)
No enoxaparin
Heparin treatment dose
Heparin prophylactic dose (subcutaneous)
No heparin
Comorbidities
Coronary Artery Disease
Cerebrovascular Accident (Stroke)
Heart Failure
Hypertension
Diabetes Mellitus
Pulmonary Disease
Renal Disease
Cirrhosis
Hepatitis
HIV
Active Cancer
Transplant
Inflammatory Bowel Disease
Rheumatologic Disease
Other Immunosuppressed State
Demographics
Age
Sex female
AFRICAN AMERICAN
ASIAN / PACIFIC ISLANDER
CAUCASIAN
MULTI-RACIAL
OTHER Race
BMI
1 2 3 4 5
1 2 3 4 5
1 2 3 4 5
Day post-intubation
1.0
0.8
0.6
0.4
0.2
0.0
Normalized predictor importance

Figure S8. Predictor importance of the subphenotype prediction models. Horizontal axis of each heatmap presents timepoint post-intubation when data were used to train the random forest-based prediction model of the worsening and recovering subphenotypes. Color intensity represents the normalized importance of a predictor in a specific prediction model.

Abbreviations: BMI=body mass index, FiO2=fraction of inspired oxygen, HIV=human immunodeficiency viruses, PaO2=partial pressure of oxygen, PBW=predicted body weight, PCO2=arterial partial pressure of carbon dioxide, P/F ratio=PaO2/FiO2 ratio, SpO2=oxygen saturation.
